# Neuroscience meets behavior: A systematic literature review on magnetic resonance imaging of the brain combined with real‐world digital phenotyping

**DOI:** 10.1002/hbm.26620

**Published:** 2024-03-04

**Authors:** Ana María Triana, Jari Saramäki, Enrico Glerean, Nicholas Mark Edward Alexander Hayward

**Affiliations:** ^1^ Department of Computer Science, School of Science Aalto University Espoo Finland; ^2^ Department of Neuroscience and Biomedical Engineering, School of Science Aalto University Espoo Finland

**Keywords:** actigraph, ambulatory assessment, diffusion tensor imaging, diffusion weighted imaging, digital phenotyping, ecological momentary assessments, EMA, fMRI, functional magnetic resonance imaging, magnetic resonance imaging, MRI, physical activity, physiology, portable device, sleep, smart monitoring, smartphone, smartwatch, structural magnetic resonance imaging, wearable

## Abstract

A primary goal of neuroscience is to understand the relationship between the brain and behavior. While magnetic resonance imaging (MRI) examines brain structure and function under controlled conditions, digital phenotyping via portable automatic devices (PAD) quantifies behavior in real‐world settings. Combining these two technologies may bridge the gap between brain imaging, physiology, and real‐time behavior, enhancing the generalizability of laboratory and clinical findings. However, the use of MRI and data from PADs outside the MRI scanner remains underexplored. Herein, we present a Preferred Reporting Items for Systematic Reviews and Meta‐Analysis systematic literature review that identifies and analyzes the current state of research on the integration of brain MRI and PADs. PubMed and Scopus were automatically searched using keywords covering various MRI techniques and PADs. Abstracts were screened to only include articles that collected MRI brain data and PAD data outside the laboratory environment. Full‐text screening was then conducted to ensure included articles combined quantitative data from MRI with data from PADs, yielding 94 selected papers for a total of *N* = 14,778 subjects. Results were reported as cross‐frequency tables between brain imaging and behavior sampling methods and patterns were identified through network analysis. Furthermore, brain maps reported in the studies were synthesized according to the measurement modalities that were used. Results demonstrate the feasibility of integrating MRI and PADs across various study designs, patient and control populations, and age groups. The majority of published literature combines functional, T1‐weighted, and diffusion weighted MRI with physical activity sensors, ecological momentary assessment via PADs, and sleep. The literature further highlights specific brain regions frequently correlated with distinct MRI‐PAD combinations. These combinations enable in‐depth studies on how physiology, brain function and behavior influence each other. Our review highlights the potential for constructing brain–behavior models that extend beyond the scanner and into real‐world contexts.

## Graphical abstract

This systematic review of 94 papers highlights increasing interest in merging magnetic resonance imaging (MRI) techniques with digital phenotyping data, reflected in growing publications since 2007. The diverse behaviors, MRI combinations, and sample characteristics studied signal a paradigm shift in brain research toward more naturalistic, real‐life contexts.

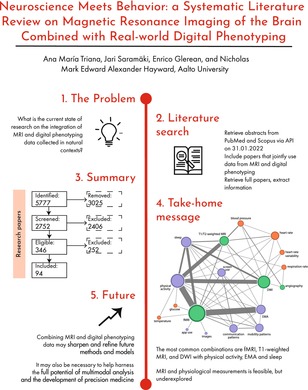

## 1 INTRODUCTION

Our cognitive and physiological systems are engaged in a complex interplay, coordinated by the brain and continuously adapting to environmental stimuli. This joint system can be approached both from the perspective of the underlying brain mechanisms and from the behavioral point of view. Technology advances, such as magnetic resonance imaging (MRI), have facilitated the noninvasive study of the brain. Different MRI techniques have been employed to investigate the brain's anatomy (Durston et al., 2001), physiology (Frahm et al., 1994), structure (Assaf & Pasternak, 2008), and function (Logothetis, 2008). However, despite the progress made in uncovering brain processes, there are still gaps in our knowledge of how the brain generates behavior (Krakauer et al., 2017). Specifically, the artificial conditions of laboratory and clinical settings can decouple subjects from the environment in which they operate (Nastase et al., 2020), making it challenging to apply laboratory results to real‐life situations. Simultaneously, the emergence of portable automatic devices (PADs) has revolutionized the measurement of human behavior and physiology in real‐world settings. PADs are compact, wearable, or handheld electronic devices that automatically collect behavioral or physiological data with minimal to no user input. They facilitate *digital phenotyping*, the quantification of human phenotypes via gadgets in situ (Jain et al., 2015; Torous et al., 2016). Examples of PADs include fitness trackers that gather data on human bodily functions (physiology) and smartphones that collect data of human interactions with the environment or of mental states via self‐reports (behavior). Although PADs significantly enhance our understanding of health‐related habits within natural settings, a critical challenge remains: unlike MRI scanners, PADs can only provide information about behavior and physiology, but are unable to relate it to intricate brain biology and function. Moreover, many PAD studies are observational, therefore providing mostly correlational information (McGowan et al., 2023; Moura et al., 2022).

The gap in integrating neurological understanding with the environmental context is highlighted by the dual technological evolution, where MRI reveals intricate details of brain function in artificial, controlled settings and PADs capture real‐life human behavior and physiology without direct insights into the underlying brain function. While each technology independently contributes valuable information, their isolated use in studies often leads to a fragmented comprehension of how human brain function relates to behavioral and physiological patterns in everyday environments. This need for a more integrative strategy resonates with prior viewpoints (Krakauer et al., 2017; Marom et al., 2009), where it is argued that the current approach of causal manipulation is insufficient for fully understanding the brain's role in behavior, much like how studying feathers alone is not enough to explain how birds fly (Marr, 1978).

Consequently, the integration of PADs in neuroscience research and clinical practice offers a promising path to bridge the gap between brain imaging and real‐world behavior, improving the generalizability of laboratory findings, and strengthening the statistical power of evidence supporting the ecological validity and clinical relevance of MRI studies. Despite these clear advantages, we found only one review paper that discusses the combined application of MRI techniques and ambulatory assessment in examining brain–behavior relationships in natural contexts (McGowan et al., 2023). In this work, the authors emphasize the necessity of frameworks for integrating MRI and PAD data for a deeper understanding of brain–behavior interactions, primarily focusing on the methods employed in analyzing combined MRI‐PAD data. Here, we report a wider range of PADs and identify the main brain areas found in the studies categorized by MRI‐PAD combination. These brain areas are analyzed in both, a descriptive summary at the level of large regions of interest (ROIs) and a coordinate‐based meta‐analysis. Consequently, to expand on the topic, in this systematic literature review, we focus on studies that jointly analyzed MRI data and information collected by PADs outside the scanner.

Our approach involved systematically searching for studies incorporating both technologies, followed by meticulous data selection, extraction, and synthesis. The results of this review provide four main contributions: (i) we characterize and summarize the selected studies, synthesizing findings on true feasibility of combining data from PADs with MRI (ii) we identify frequently used MRI‐device combinations and identify underexplored MRI‐PAD pairings, (iii) we show the brain areas yielding statistically significant results in those studies, and (iv) we discuss trends and open issues to inform future research.

The structure of the review is as follows. In Section 2, we outline the methodology. In Sections 3.2, 3.7, we characterize and synthesize the selected studies. In Section 3.8, we examine the concurrent use of MRI and devices in the studies, both in average and over time. In Section 3.9, we show the statistically significant brain areas identified in the studies for each MRI‐device combination. Finally, in Section 4, we discuss identified trends, open issues, and summarize our findings.

## 2 MATERIALS AND METHODS

We conducted a systematic literature review using two databases, PubMed and Scopus. Initially, we developed a protocol for systematic data retrieval and analysis, which was preregistered in the Open Science Framework (Triana et al., 2022). Following this protocol, we analyzed and synthesized the data to provide a summary of relevant information in this research field.

### 2.1 Objective and research question

This systematic literature review aims to provide a comprehensive understanding of how scientists and clinicians have employed PADs in conjunction with MRI. We ask two questions: (i) What is the current state of research on the integration of MRI and PAD data collected in natural contexts? and (ii) What are the characteristics of these studies?

### 2.2 Search strategy and selection process

To ensure a comprehensive search, we designed a search string based on two keywords extracted from two sets listed in Table 1. Set “A” includes terms commonly used in research studies analyzing brain signals using MRI techniques (e.g., functional MRI [fMRI], diffusion tensor imaging, etc.), and set “B” comprises keywords typically used in digital phenotyping papers (e.g., actigraph, smartphone, etc.). Each string is a combination of a keyword from set “A” and a keyword from set “B” using the operator AND (e.g., “fMRI” AND “sleep”).

**TABLE 1:** Keywords and sets used to design the search string.

Set	Keywords
A	Structural magnetic resonance imaging, cortical thickness, diffusion magnetic resonance imaging, diffusion tensor imaging, fractional anisotropy, magnetic resonance venography, magnetic resonance angiography, functional magnetic resonance imaging, functional connectivity, ReHo, representational similarity analysis, intersubject correlation, statistical parametric mapping, FSL, freesurfer, neurosynth, neurovault, Amplitude of Low Frequency Fluctuations, blood oxygen level dependent, functional connectivity density, resting state fMRI, task fMRI, resting‐state functional magnetic resonance imaging, task functional magnetic resonance imaging
B	Accelerometer, apps, phone battery, bluetooth, inferred conversation, smartphone light sensor, global positioning system, smartphone keyboard, smartphone screen, smartphone microphone, smartwatch, actigraph, oura, ecological momentary assessments, experience sampling method, environmental audio, heart rate, sleep duration, smartphone use, calls, short message service, indoor mobility, outdoor mobility, footsteps, calories, social interaction, physical activity, screen brightness, mobile health, medical informatics, smartphone, digital phenotype, passive data, active data, wearables, personal sensing, objective sensor data, early warning signals, smartphone apps, GPS, EMA, ESM, phone, breathing rate, respiratory rate, heart rate variation, heart rate variability, blood pressure, oximetry, oxygen saturation, pulse rate, activity tracker, smart monitoring, eye tracking, mHealth, ambulatory assessment

Once all the possible combinations were established, we automatically retrieved papers from the two databases. These databases were chosen based on their coverage of scientific publications and their APIs, which allowed us to automate the search process. Each combination was treated as a query, generating a list of unique identifiers (PMID or Scopus ID) for the matched papers. Subsequently, we fetched basic research article information, including title, abstract, publication date, DOI, and keywords. Duplicate entries were removed, and the gathered data were systematically organized.

An electronic search was performed on January 31, 2022, covering the last 22 years (2000–2022) of literature. We searched for pairs of terms (“set A keyword” AND “set B keyword”) in the title and abstract. Only studies in English were included. Due to database API characteristics, we restricted the document types to journal articles, clinical studies, and congress in PubMed and to article and conference papers in Scopus.

### 2.3 Eligibility criteria

Primary research studies were selected if they met the following inclusion criteria: (i) the study analyzed data from human subjects; (ii) the study analyzed data collected with PADs in everyday‐life conditions (i.e., not in clinical or laboratory settings), and (iii) the study analyzed MRI data in combination with data from PADs. Papers were excluded if: (i) they were literature reviews or protocols; (ii) they did not clearly state what technology/device is employed (e.g., a study that administered questionnaires but did not mention if they are paper‐based or electronically delivered); (iii) they mentioned a device but did not use the collected data (e.g., a paper where actigraph data were collected but not analyzed), (iv) researchers employed the device under clinical or laboratory settings (e.g., an intervention study where heart rate was monitored using a smartwatch during staff‐supervised sessions or a study where heart rate was measured only while scanning), and (v) MRI or device data were used to verify states or as a back‐up option (e.g., data from an actigraph that was only used to verify sleep‐deprived nights before a scanning session, but not analyzed). Note that standard computers are not considered as PADs. Moreover, for brain imaging, our scope is limited to MRI conducted in a laboratory or clinical setting, and therefore we also exclude EEG devices even when worn outside the laboratory.

### 2.4 Screening process

The screening process was divided in two parts, abstract screening and full‐text inclusion. Three study authors (NMEAH, EG, and AMT) independently screened all the titles and abstracts. Keywords from the query that yielded the paper were also available at this stage to provide additional information. Each author assigned a score of 1 for inclusion, −1 for exclusion, or 0 for uncertainty. After all abstracts were screened, we calculated an agreement score to determine paper inclusion or exclusion. A paper advanced to the next stage if two authors assigned it a score of 0 or 1. Authors were blinded to each other's decisions during the abstract screening process.

Next, full‐text documents of the selected papers were obtained to determine their final inclusion status. One reviewer (AMT) screened the methods section of each paper to ensure the research papers combined MRI and PAD data. Similarly to the abstract screening, the author assigned a score of 1 for inclusion, −1 for exclusion, or 0 for uncertainty, in which case, NMEAH and EG provided their independent opinions to reach a consensus.

### 2.5 Data extraction

At this stage, we read the full texts of the selected papers. For each paper, we extracted information about the characteristics of the samples, data quality, study design, type and functionality of the device, how long the device was worn, MRI technique and stimulus, analysis methods, reported brain areas and coordinates, question of interest, findings, and conclusions.

### 2.6 Graph analysis

Based on categorized data, we created a network to identify the most common combinations of MRI and devices employed in the selected sample. In this network, nodes represent MRI techniques and PADs (labeled according to their functions). The weight of a link indicates the number of papers using the respective techniques or devices. We organized PADs into two color‐coded sets—physiology and behavior—based on what they measure. In this context, physiological measurements refer to the quantification of human bodily functions, such as heart rate, while behavioral measurements cover a range of human actions, interactions, and self‐reported states, including sleep patterns and response to questionnaires. Finally, we used the algorithm of Ahn et al. (2010) to detect link communities for identifying close relationships between MRI techniques and devices. This algorithm detects sets of closely related links (link communities), which allows nodes to belong to multiple communities at the same time.

### 2.7 Brain area visualization

For each of the included studies, we mapped the reported list of statistically significant brain areas into the AALv3 atlas (Rolls et al., 2020). First, we conducted automated string matching, followed by manual quality control. Similar to NeuroSynth (Yarkoni et al., 2011) (where peak coordinates from activations are converted into spheres), this approach allows us to generate a brain mask for each study, labeling reported ROIs as significant with 1 s and nonsignificant areas with zeros. Since not all studies reported coordinates, we opted for a descriptive statistics approach using ROIs. Our goal was to graphically visualize the areas that were most frequently reported across studies, presenting frequency counts of the brain areas reported for each MRI‐PAD combination. In addition, we ran a meta‐analysis using NIMARE (Salo et al., 2022; Salo et al., 2023) for those few cases where enough coordinates are reported. For both cases, we limited the synthesis of brain maps to T1‐weighted and fMRI studies due to the complexity of remapping white matter tracts to the standard MNI space.

### 2.8 Data and software availability

The code developed for this literature review is available at Zenodo (Triana et al., 2023).

## 3 RESULTS

### 3.1 Study selection

**FIGURE 1:**
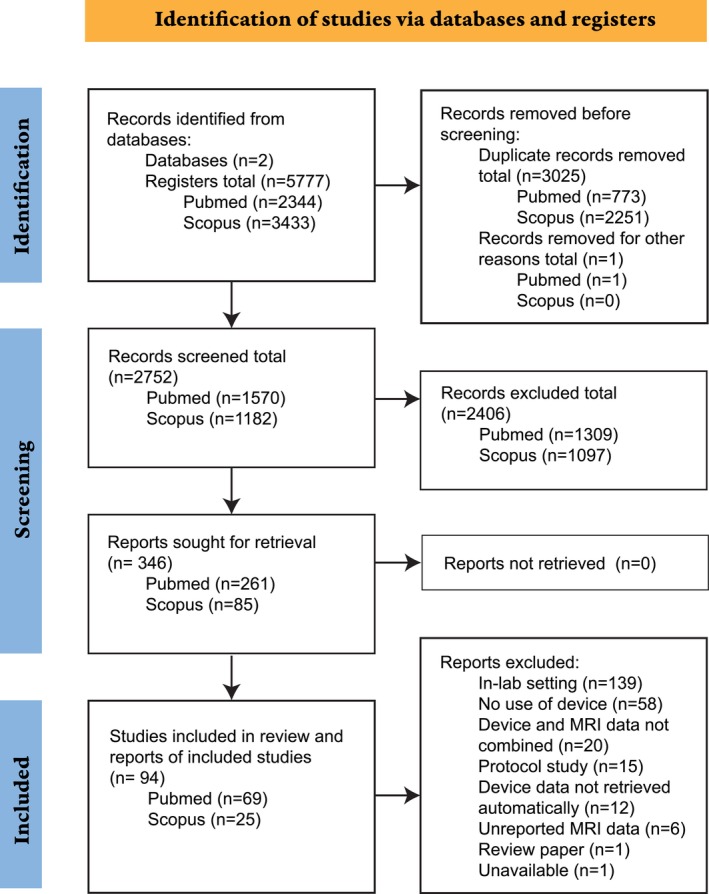
Preferred Reporting Items for Systematic Reviews and Meta‐Analysis (PRISMA) 2020 guidelines flow chart showing the search strategy and selection process for this review.

Figure 1 shows the diagram of the selection process, according to the Preferred Reporting Items for Systematic Reviews and Meta‐Analysis statement (Moher et al., 2009). First, 5777 registers were automatically retrieved from two databases (PubMed and Scopus). Of these, we discarded 3024 duplicated papers and 1 book. Next, we screened the abstracts of the remaining 2752 papers and agreed to exclude 2406, selecting 346 papers. Of these, 252 papers were excluded because they did not comply with the inclusion criteria, resulting in 94 studies being selected.

To calculate interrater agreement, we used the κ Fleiss statistic as it is the most commonly used analysis technique in systematic reviews (Belur et al., 2021). We also report the overall rater agreement in percentage. When looking at the interrater agreement, the value of κ Fleiss statistic was 0.283 which corresponds to “Fair agreement” (Belur et al., 2021). The percentage of agreement, however, (68.6%) indicates a substantial agreement. This discrepancy between the low value of κ and the higher percentage of agreement is common when the rating distribution is skewed as is the case in this literature review (see Supplementary Figure S1).

### 3.2 Characterization of the selected studies

The reviewed studies and their main characteristics are listed in Table 2. An extended version of this table can be found in Supplementary Table S1.

**TABLE 2:** Summary of reviewed studies.

ID	Authors	Title	MRI technique	Device (by function)	Device
P01	Hamer et al. (2018)	Association of objectively measured physical activity with brain structure: UK Biobank study	T1/T2‐weighted MRI	Physical activity	Accelerometer
P02	Makizako et al. (2015)	Moderate‐intensity physical activity, hippocampal volume, and memory in older adults with mild cognitive impairment	T1/T2‐weighted MRI	Physical activity	Accelerometer, step counter
P03	Pindus et al. (2020)	Opposing associations between sedentary time and decision‐making competence in young adults revealed by functional connectivity in the dorsal attention network	fMRI	Physical activity	Accelerometer
P04	Bakker et al. (2019)	From laboratory to life: associating brain reward processing with real‐life motivated behavior and symptoms of depression in non‐help‐seeking young adults	fMRI	EMA/ESM (mood, daily events and activities)	beeper
P05	Seidel et al. (2018)	The real‐life costs of emotion regulation in anorexia nervosa: a combined ecological momentary assessment and fMRI study	fMRI	EMA/ESM (disorder‐related rumination and affect)	Smartphone
P06	Wilson et al. (2013)	Integrating ecological momentary assessment and functional brain imaging methods: new avenues for studying and treating tobacco dependence	fMRI	EMA/ESM (smoking habits, triggers, craving, nicotine dependence, affective valence, and arousal)	Smartphone
P07	Collip et al. (2013)	Hippocampal volume as marker of daily life stress sensitivity in psychosis	T1/T2‐weighted MRI	EMA/ESM (emotional stress reactivity and negative affect)	Wristwatch
P08	Karch et al. (2019)	Identifying predictors of within‐person variance in MRI‐based brain volume estimates	T1/T2‐weighted MRI	Physical activity, sleep	Wristwatch
P09	Bengtsson et al. (2020)	Autonomic modulation networks in schizophrenia: The relationship between heart rate variability and functional and structural connectivity in the brain	DWI, fMRI	Heart rate, heart rate variability	Heart rate monitor
P10	Varma et al. (2015)	Low‐intensity daily walking activity is associated with hippocampal volume in older adults	T1/T2‐weighted MRI	Physical activity	Accelerometer
P11	Voss et al. (2016)	Fitness, but not physical activity, is related to functional integrity of brain networks associated with aging	fMRI	Physical activity	Accelerometer
P12	Ruotsalainen et al. (2020)	Physical activity, aerobic fitness, and brain white matter: Their role for executive functions in adolescence	DWI	Physical activity	Accelerometer
P13	Bracht et al. (2018)	Physical activity is associated with left corticospinal tract microstructure in bipolar depression	DWI	Physical activity	Accelerometer
P14	Bracht et al. (2016)	Myelination of the right parahippocampal cingulum is associated with physical activity in young healthy adults	DWI	Physical activity	Accelerometer
P15	Tian et al. (2015)	Objective measures of physical activity, white matter integrity and cognitive status in adults over age 80	DWI	Physical activity	Accelerometer, wristwatch
P16	Burzynska et al. (2014)	Physical activity and cardiorespiratory fitness are beneficial for white matter in low‐fit older adults	DWI, T1/T2‐weighted MRI	Physical activity	Accelerometer
P17	Soehner et al. (2018)	Cognitive control under stressful conditions in transitional age youth with bipolar disorder: Diagnostic and sleep‐related differences in fronto‐limbic activation patterns	fMRI	Sleep	Accelerometer, wristwatch
P18	Tashjian et al. (2017)	Neural connectivity moderates the association between sleep and impulsivity in adolescents	fMRI	Sleep	Accelerometer, smartphone
P19	Khalsa et al. (2016)	Variability in Cumulative Habitual Sleep Duration Predicts Waking Functional Connectivity	fMRI	Sleep	Accelerometer
P20	Huckins et al. (2019)	Fusing Mobile Phone Sensing and Brain Imaging to Assess Depression in College Students	fMRI	Communication patterns, EMA/ESM (PHQ‐4), mobility patterns, screen use	Smartphone
P21	Kimura et al. (2013)	Correlation between moderate daily physical activity and neurocognitive variability in healthy elderly people	fMRI	Physical activity	Step counter
P22	Ellingson et al. (2012)	Physical activity, sustained sedentary behavior, and pain modulation in women with fibromyalgia	fMRI	Physical activity	Accelerometer
P23	McLoughlin et al. (2011)	The relationship between physical activity and brain responses to pain in fibromyalgia	fMRI	Physical activity	Accelerometer
P24	Szabo‐Reed et al. (2020)	Modeling interactions between brain function, diet adherence behaviors, and weight loss success	fMRI	Physical activity	Step counter
P25	Whelan et al. (2017)	Brain Activation in Response to Personalized Behavioral and Physiological Feedback From Self‐Monitoring Technology: Pilot Study	fMRI	Glucose, physical activity	Accelerometer
P26	Veldsman et al. (2017)	Physical Activity After Stroke Is Associated With Increased Interhemispheric Connectivity of the Dorsal Attention Network	fMRI	Physical activity, temperature	Accelerometer, wristwatch
P27	Cascio et al. (2016)	Self‐affirmation activates brain systems associated with self‐related processing and reward and is reinforced by future orientation	fMRI	Physical activity	Accelerometer
P28	Khalsa et al. (2017)	Habitual sleep durations and subjective sleep quality predict white matter differences in the human brain	DWI	Sleep	Accelerometer
P29	Ahmed et al. (2017)	Energy expenditure in frontotemporal dementia: a behavioral and imaging study	T1/T2‐weighted MRI	Heart rate, physical activity	Heart rate monitor
P30	Schwartz et al. (2019)	Resting‐state functional connectivity and inflexibility of daily emotions in major depression	fMRI	EMA/ESM (depression symptoms, and participants' feelings and thoughts)	Smartphone
P31	Dlamini et al. (2017)	Nocturnal oxyhemoglobin desaturation and arteriopathy in a pediatric sickle cell disease cohort	angiography	Respiration rate	Pulse oxymeter
P32	Sreekumar et al. (2018)	The experience of vivid autobiographical reminiscence is supported by subjective content representations in the precuneus	fMRI	Images	Smartphone
P33	Vreeker et al. (2021)	Genetic analysis of activity, brain and behavioral associations in extended families with heavy genetic loading for bipolar disorder	T1/T2‐weighted MRI	Physical activity, sleep	Accelerometer
P34	Burzynska et al. (2017)	White matter integrity declined over 6 months, but Dance Intervention Improved Integrity of the Fornix of Older Adults	DWI	Physical activity	Accelerometer
P35	Verkooijen et al. (2017)	The association of sleep and physical activity with integrity of white matter microstructure in bipolar disorder patients and healthy controls	DWI	Physical activity, sleep	Accelerometer
P36	Docx et al. (2017)	White matter microstructure and volitional motor activity in schizophrenia: A diffusion kurtosis imaging study	DWI	Physical activity	Accelerometer
P37	Celle et al. (2012)	Association between ambulatory 24‐h blood pressure levels and brain volume reduction: a cross‐sectional elderly population‐based study	T1/T2‐weighted MRI	Blood pressure	Blood pressure monitor
P38	Barnden et al. (2011)	A brain MRI study of chronic fatigue syndrome: evidence of brainstem dysfunction and altered homeostasis	T1/T2‐weighted MRI	Blood pressure, heart rate	Blood pressure monitor
P39	McKenna et al. (2014)	Associations between circadian activity rhythms and functional brain abnormalities among euthymic bipolar patients: a preliminary study	fMRI	Physical activity, sleep	Accelerometer, wristwatch
P40	Holm et al. (2009)	Reward‐related brain function and sleep in pre/early pubertal and mid/late pubertal adolescents	fMRI	Sleep	Accelerometer
P41	Miedl et al. (2020)	Neural Processing During Fear Extinction Predicts Intrusive Memories	fMRI	EMA/ESM (intrusive memories)	Smartphone
P42	Janes et al. (2019)	Quitting starts in the brain: a randomized controlled trial of app‐based mindfulness shows decreases in neural responses to smoking cues that predict reductions in smoking	fMRI	EMA/ESM (smoking habits, triggers, craving, and mindfulness practices)	Smartphone
P43	Tarumi et al. (2019)	Ambulatory pulse pressure, brain neuronal fiber integrity, and cerebral blood flow in older adults	DWI, angiography	Blood pressure	Blood pressure monitor
P44	Lakhani et al. (2017)	Hemispheric asymmetry in myelin after stroke is related to motor impairment and function	T1/T2‐weighted MRI	Physical activity	Accelerometer
P45	Kim et al. (2020)	White Matter Integrity is Associated With the Amount of Physical Activity in Older Adults With Super‐aging	DWI	Physical activity, sleep	Accelerometer, wristwatch
P46	Webb et al. (2021)	Mind‐Wandering in Adolescents Predicts Worse Affect and is Linked to Aberrant Default Mode Network‐Salience Network Connectivity	fMRI	EMA/ESM (positive and negative affect, mind‐wandering, current activity, social context, rumination)	Smartphone
P47	Fleming, Piro‐Gambetti, Bazydlo, et al. (2021)	Sleep and White Matter in Adults with Down Syndrome	DWI	Sleep	Accelerometer
P48	Machida et al. (2022)	Objectively measured intensity‐specific physical activity and hippocampal volume among community‐dwelling older adults	T1/T2‐weighted MRI	Physical activity	Accelerometer
P49	J. L. Smith et al. (2021)	Impact of App‐Delivered Mindfulness Meditation on Functional Connectivity, Mental Health, and Sleep Disturbances Among Physician Assistant Students: Randomized, Wait‐list Controlled Pilot Study	fMRI	App use	Smartphone
P50	Wei et al. (2021)	White Matter Integrity Underlies the Physical‐Cognitive Correlations in Subjective Cognitive Decline	DWI	Physical activity	Accelerometer
P51	Wisman‐van der Teen et al. (2022)	Exploring the association between social behavior, trust, and its neural correlates in first episode psychosis patients and in individuals at clinical high risk for psychosis	fMRI	EMA/ESM (activity, positive and negative affect, psychotic symptoms, and social reactivity)	Beeper
P52	Thomas et al. (2021)	Digital sleep measures and white matter health in the Framingham Heart Study	DWI	Respiration rate, sleep	Accelerometer
P53	Moy et al. (2021)	Co‐occurrence of pain and dyspnea in Veterans with COPD: Relationship to functional status and a pilot study of neural correlates using structural and functional magnetic resonance imaging	T1/T2‐weighted MRI, fMRI	Physical activity	Step counter
P54	Horstman et al. (2022)	Fathers' Involvement in Early Childcare is Associated with Amygdala Resting‐State Connectivity	fMRI	EMA/ESM (proximity and interaction with the baby)	Smartphone
P55	Domingos et al. (2021)	Free‐Living Physical Activity Measured With a Wearable Device Is Associated With Larger Hippocampus Volume and Greater Functional Connectivity in Healthy Older Adults: An Observational, Cross‐Sectional Study in Northern Portugal	T1/T2‐weighted MRI, fMRI	Physical activity	Wristwatch
P56	Balbim et al. (2021)	The Impact of the BAILAMOSâ„¢ Dance Program on Brain Functional Connectivity and Cognition in Older Latino Adults: A Pilot Study	fMRI	Physical activity	Accelerometer
P57	Ruotsalainen et al. (2021)	Physical activity and aerobic fitness in relation to local and interhemispheric functional connectivity in adolescents' brains	fMRI	Physical activity	Accelerometer
P58	Provenzano et al. (2021)	Inflexibly sustained negative affect and rumination independently link default mode network efficiency to subclinical depressive symptoms	fMRI	EMA/ESM (positive and negative emotional experience)	Smartphone
P59	Morris et al. (2022)	Resting state functional connectivity provides mechanistic predictions of future changes in sedentary behavior	fMRI	Physical activity	Accelerometer
P60	Wolf et al. (2020)	The Impact of Age on the Association Between Physical Activity and White Matter Integrity in Cognitively Healthy Older Adults	DWI	Physical activity	Accelerometer, wristwatch
P61	Rabin et al. (2019)	Associations of Physical Activity and β‐Amyloid With Longitudinal Cognition and Neurodegeneration in Clinically Normal Older Adults	T1/T2‐weighted MRI	Physical activity	Step counter
P62	Zhang et al. (2020)	Sleep inconsistency between weekends and weekdays is associated with changes in brain function during task and rest	fMRI	Sleep	Accelerometer
P63	Fleming, Piro‐Gambetti, Patrick, et al. (2021)	Physical activity and cognitive and imaging biomarkers of Alzheimer's disease in down syndrome	DWI	Physical activity	Accelerometer
P64	Sanders et al. (2021)	Linking objective measures of physical activity and capability with brain structure in healthy community dwelling older adults	DWI, T1/T2‐weighted MRI	Physical activity	Step counter
P65	Dion et al. (2021)	Functional connectivity of key resting state networks and objectively measured physical activity in older adults with joint pain: A pilot study	fMRI	Physical activity	Accelerometer
P66	Kalafatakis et al. (2021)	Glucocorticoid ultradian rhythmicity differentially regulates mood and resting state networks in the human brain: A randomized controlled clinical trial	fMRI	EMA/ESM (reactivity, feeling of wellbeing, and mood)	Smartphone
P67	Chang et al. (2022)	Functional Connectivity, Physical Activity, and Neurocognitive Performances in Patients with Vascular Cognitive Impairment, No Dementia	fMRI	Physical activity	Wristwatch
P68	Gogniat et al. (2021)	The Relations Between Physical Activity Level, Executive Function, and White Matter Microstructure in Older Adults	DWI	Physical activity	Accelerometer
P69	Hua, Trull, Merrill, Tidwell, and Kerns (2021)	Functional connectivity between the ventral anterior cingulate and amygdala during implicit emotional conflict regulation and daily‐life emotion dysregulation	fMRI	EMA/ESM (mood, emotion dysregulation)	Smartphone
S01	Falck et al. (2020)	Not Just for Joints: The Associations of Moderate‐to‐Vigorous Physical Activity and Sedentary Behavior with Brain Cortical Thickness	T1/T2‐weighted MRI	Physical activity	Accelerometer
S02	Rodriguez‐Ayllon et al. (2020)	Physical Activity, Sedentary Behavior, and White Matter Microstructure in Children with Overweight or Obesity	DWI	Physical activity	Accelerometer
S03	Michielse et al. (2020)	White matter microstructure and network‐connectivity in emerging adults with subclinical psychotic experiences	DWI	EMA/ESM (subclinical psychotic experiences)	Beeper
S04	Durning et al. (2015)	Relationship of neuroimaging to typical sleep times during a clinical reasoning task: A pilot study	fMRI	Sleep	Accelerometer
S05	Servaas et al. (2019)	Rigidity in motor behavior and brain functioning in patients with schizophrenia and high levels of apathy	fMRI	Physical activity	Accelerometer
S06	Culbreth et al. (2020)	Effort, Avolition, and Motivational Experience in Schizophrenia: Analysis of Behavioral and Neuroimaging Data With Relationships to Daily Motivational Experience	fMRI	EMA/ESM (current activities, level of interest and enjoyment)	Smartphone
S07	Tashjian et al. (2018)	Sleep quality and adolescent default mode network connectivity	fMRI	Sleep	Accelerometer
S08	Prakash et al. (2011)	Physical activity associated with increased resting‐state functional connectivity in multiple sclerosis	fMRI	Physical activity	Accelerometer
S09	Kokotilo et al. (2010)	Greater Activation of Secondary Motor Areas Is Related to Less Arm Use After Stroke	fMRI	Physical activity	Accelerometer
S10	Kluge et al. (2018)	Combining accelerometery, ecological momentary assessment and neuroimaging to study apathy in patients with schizophrenia	fMRI	EMA/ESM (apathy and diminished expression), physical activity	Accelerometer, beeper
S11	Lopez et al. (2016)	Motivational and neural correlates of self‐control of eating: A combined neuroimaging and experience sampling study in dieting female college students	fMRI	EMA/ESM (eating behaviors)	Smartphone
S12	Li et al. (2020)	Low Hippocampal Dentate Gyrus Volume Associated With Hypertension‐Related Cognitive Impairment	T1/T2‐weighted MRI	Blood pressure	Blood pressure monitor
S13	A. R. Smith et al. (2019)	Advancing clinical neuroscience through enhanced tools: Pediatric social anxiety as an example	fMRI	EMA/ESM (emotions, momentary effect and recent interactions with peers)	Smartphone
S14	Moran et al. (2019)	From Neuroimaging to Daily Functioning: A Multimethod Analysis of Reward Anticipation in People With Schizophrenia	fMRI	EMA/ESM (anticipated motivation and pleasure in daily activities)	Smartphone
S15	Kocevska et al. (2019)	The prospective association of objectively measured sleep and cerebral white matter microstructure in middle‐aged and older persons	DWI	Sleep	Accelerometer, wristwatch
S16	Eisenberger et al. (2007)	Functional Magnetic Resonance Imaging Responses Relate to Differences in Real‐World Social Experience	fMRI	EMA/ESM (neuroticism, social anxiety, rejection sensitivity)	Beeper
S17	Berkman et al. (2011)	In the trenches of real‐world self‐control: Neural correlates of breaking the link between craving and smoking	fMRI	EMA/ESM (smoking consumption, craving, and mood)	Smartphone
S18	Obuchi et al. (2020)	Predicting brain functional connectivity using mobile sensing	fMRI	Communication patterns, mobility patterns, physical activity, screen use, sleep	Smartphone
S19	Hisamatsu et al. (2021)	Association of self‐measured home, ambulatory, and strictly measured office blood pressure and their variability with intracranial arterial stenosis	angiography	Blood pressure	Blood pressure monitor
S20	Sequeira et al. (2021)	From scanners to cell phones: Neural and real‐world responses to social evaluation in adolescent girls	fMRI	EMA/ESM (social interactions and emotional responses)	Smartphone
S21	Krönke et al. (2020)	Functional connectivity in a triple‐network saliency model is associated with real‐life self‐control	fMRI	EMA/ESM (self‐control)	Smartphone
S22	Krönke et al. (2021)	Real‐Life Self‐Control is Predicted by Parietal Activity During Preference Decision‐Making: A Brain Decoding Analysis	fMRI	EMA/ESM (self‐control)	Smartphone
S23	Jalbrzikowski et al. (2021)	Associations between brain structure and sleep patterns across adolescent development	T1/T2‐weighted MRI	Sleep	Accelerometer
S24	Hua, Trull, Merrill, Myers, et al. (2021)	Daily‐Life Negative Affect in Emotional Distress Disorders Associated with Altered Frontoinsular Emotion Regulation Activation and Cortical Gyrification	T1/T2‐weighted MRI, fMRI	EMA/ESM (negative affect)	Smartphone
S25	Lydon‐Staley et al. (2019)	Repetitive negative thinking in daily life and functional connectivity among default mode, fronto‐parietal, and salience networks	fMRI	EMA/ESM (mood and repetitive negative thinking)	Smartphone

Table 3 shows summary information including type, patient inclusion, and number of diagnoses included in each study sample. It also provides an overview of the papers' data collection processes, showing that while researchers primarily collected data for their specific studies, reanalysis of existing data (reused data) is also a common practice.

**TABLE 3:** Summary of study characteristics. The summary includes the research design type, instances where researchers reanalyzed data from existing datasets, the number of studies involving patients, and the variety of diagnoses included.

	*N*	Percentage
**Study type**	94	100
Cohort	3	3.2
Controlled‐trial	8	8.5
Cross sectional	77	81.9
Intervention	2	2.1
Longitudinal	4	4.3
**Data source**	**94**	**100**
Reused data	45	47.9
First‐use data	49	52.1
**Patient inclusion**	**94**	**100**
Patients included	41	43.6
Patients not included	53	56.4
**Number of diagnoses included**	**41**	**100**
1	38	92.7
2	2	4.9
3	1	2.4

### 3.3 Sample size and age

**FIGURE 2:**
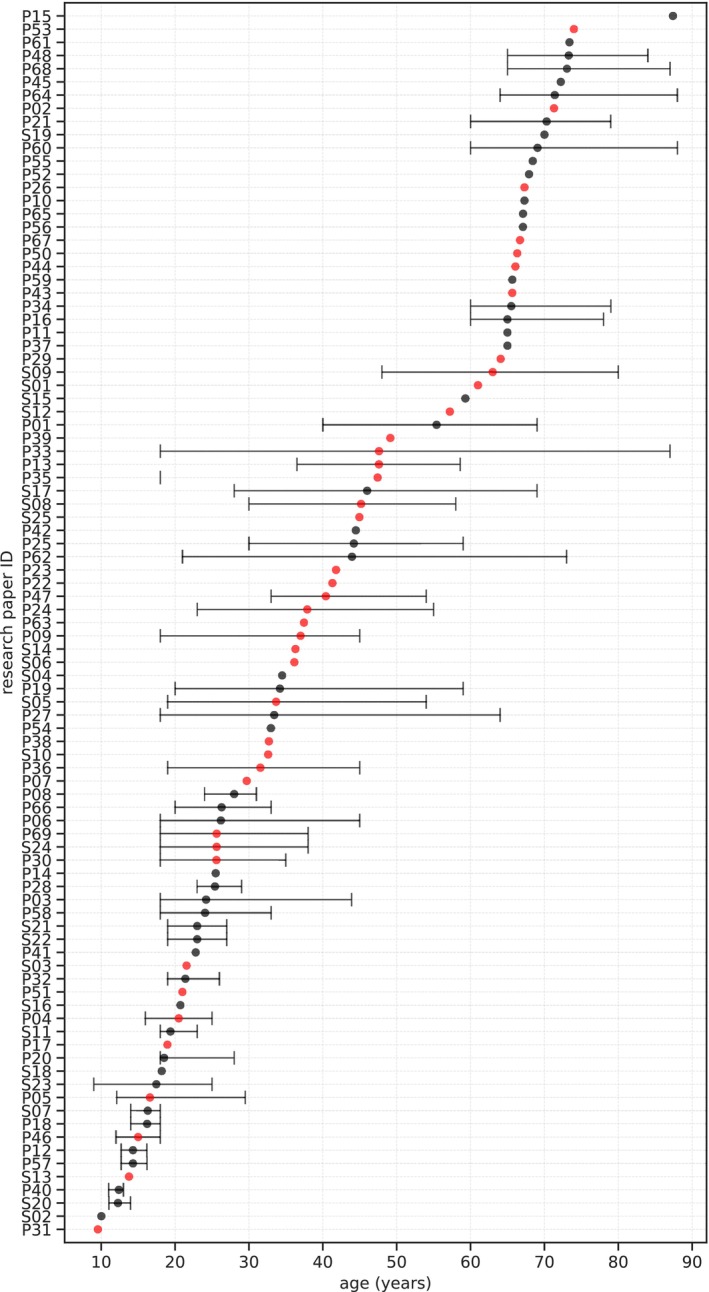
Sample mean age and age distribution of studies included in the systematic review. The study (y‐axis) was ordered by the mean age (dot) from each study. If the mean was not available, the median was used and plotted. If neither the mean nor the median were available, the average between the minimum and maximum were used. Red represents papers that included patients.

Included studies vary largely in the age of participants, which ranges from 9 to 88 years (Figure 2), irrespective of whether patients were included or not. There are no apparent gaps within this range, demonstrating a broad age representation.

**FIGURE 3:**
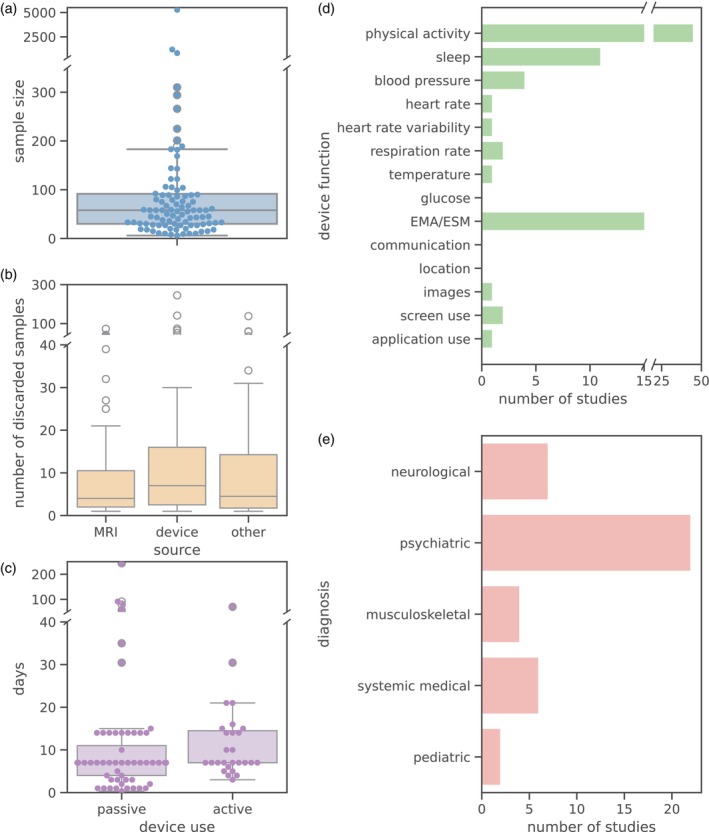
Characteristics of the studies included in the systematic review. (a) Distribution of sample sizes in the reviewed papers. (b) Distribution of excluded subjects according to the source of the issue, which could be related to magnetic resonance imaging (MRI), the device and its data collection, or other factors (e.g., unknown medical problems or phobias). (c) Number of days during which a device was used to collect data. The devices are organized into two groups based on the level of interaction required from the subject: active (some interaction is needed, such as answering a question) or passive (no interaction is required, such as heart rate measurements). (d) Number of studies employing a device based on its function to measure specific human behavior or physiological variables. (e) Diagnoses investigated in the included study sample. The studied illnesses were grouped according to their type. The number of studies that investigate an illness according to its group is shown.

On average, the studies included 157 participants. However, given the influence of extreme values (minimum sample size: 6, maximum sample size: 5272), the median of 58 participants may be a more informative measure, as can be seen in the sample size distribution (see Figure 3a).

### 3.4 Illnesses targeted

Diagnosed subjects were involved in 41 studies (40.4% of the total papers), with 92.7% of the studies featuring only one diagnosis (Table 3). Notably, three papers (7.3%) incorporated multiple diagnosis groups (ranging from two to five). The reported illnesses were classified into five categories, as shown in Figure 3e. Neurological illnesses included cognitive decline, cognitive impairment, Alzheimer's, dementia, and multiple sclerosis. Psychiatric disorders encompassed anxiety, depression, bipolar disorder, anorexia, psychoaffective, emotional distress disorder, psychosis, and schizophrenia. Musculoskeletal illnesses grouped chronic fatigue syndrome, fibromyalgia, and knee osteoarthritis. Systemic medical diseases covered hypertension, obesity, stroke, and chronic obstructive pulmonary disease. Finally, pediatric illnesses included sickle cell disease and Down syndrome.

### 3.5 Data quality

Figure 3b displays the distribution of excluded subjects according to three categories (MRI, device, and others). The median number of subjects with discarded data due to device‐related issues is slightly larger than the median number of subjects discarded due to MRI issues or other problems, such as medical illness or phobias.

### 3.6 MRI techniques

fMRI is the main MRI technique employed in the reviewed studies, followed by diffusion‐weighted imaging (DWI), T1/T2‐weighted MRI, and to a lesser extent, angiography. Task‐based fMRI is a more commonly employed technique than resting‐state fMRI (Table 4).

**TABLE 4:** Summary of employed MRI techniques and devices.

	*N*	Percentage
**MRI technique** ^a^	**101**	**100**
Diffusion	22	21.8
T1/T2‐weighted	20	19.8
Functional	56	55.4
Angiography	3	3
**fMRI technique** ^b^	**57**	**100**
Resting‐state	25	43.9
Task	32	56.1
**Device**	**94**	**100**
Accelerometer	39	41.5
Beeper	5	5.3
Wristwatch	9	9.6
Smartphone	25	26.6
Step counter	6	6.4
Other^c^	10	10.6

^a^ Some papers use more than one technique.

^b^ One study used both, task and resting‐state.

^c^ Other types include cuffs, glucose‐meters, etc

### 3.7 Portable automatic devices

Table 4 shows the most common PADs used in the reviewed papers. Accelerometers are the primary choice for portable data collection, followed by smartphones and other devices, such as cuffs and glucose meters. Beepers, wristwatches, and step counters were also employed, albeit less frequently. The devices can be further categorized by their function or the variable they measure. Figure 3d shows that researchers mainly use PADs to measure physical activity and ask customized questions (from now on referred as EMA/ESM). Variables such as sleep, blood pressure, respiration rate, and screen use have also drawn some interest. While variables like glucose, communication patterns, and GPS location have been used only once across the reviewed sample, these studies showcase the feasibility of recording such data with PADs. Further, most of the studies employed the devices for 10 days or less, regardless of whether they collected the data passively (i.e., with no use input) or actively (i.e., with user input). Four studies employed the devices during several months, mostly using passive data collection (see Figure 3c).

### 3.8 MRI and device combinations

**FIGURE 4:**
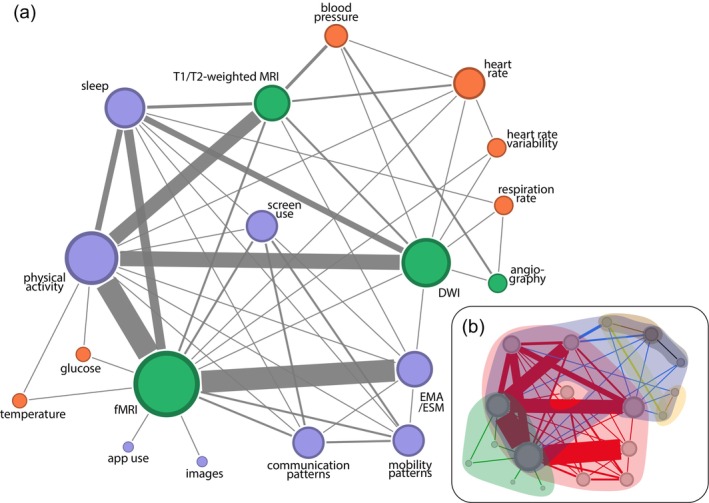
Network of co‐occurrences between portable automatic devices and magnetic resonance imaging (MRI) techniques. Link thickness represents the frequency of a particular combination in the selected research papers, with thicker links indicating higher frequency. Node size corresponds to the number of papers using a specific device or technique. (a) Nodes are color‐coded based on category: MRI (green), physiology (orange), and behavior (purple). (b) Link communities and node overlaps in the network. Links are colored according to the detected link communities that are also indicated by the shaded areas. Node positions are the same as shown in (a)).

To understand how different combinations of MRI techniques and devices were employed in the selected studies, we constructed a co‐occurrence network where nodes represent techniques or devices. The weight of the link between two nodes was determined by the number of papers combining the respective techniques or devices. Figure 4a shows the computed network (numeric values available in Supplementary Table S2).

The network visualization reveals that the majority of published literature combines fMRI, T1/T2‐weighted MRI, and DWI with physical activity sensors, EMA/ESM, and sleep. In contrast, angiography and some physiological variables (respiration rate, heart rate variability, glucose, and temperature) are less frequently employed.

Within the cluster of frequently used data sources, physical activity emerges as the most commonly measured behavioral variable. It is employed simultaneously even with other behavioral and physiological variables, most frequently with sleep and to a lesser extent with screen use, communication patterns, mobility patterns, EMA/ESM, glucose, temperature, and heart rate.

fMRI is the most frequently employed MRI technique, predominantly combined with physical activity, sleep, and EMA/ESM. fMRI is also paired with devices capable of measuring glucose, temperature, app usage, images, communication and mobility patterns, screen use, heart rate, and heart rate variability. However, fMRI data are seldom merged with physiological signals. Instead, DWI is preferred with physiological data. Yet, regarding behavioral data, the use of DWI is primarily limited to physical activity and sleep.

Among the combinations of physiological signals and MRI techniques, blood pressure with T1/T2‐weighted MRI is a particularly noteworthy combination, indicating an interest in the relationship between cardiovascular measurements and brain structure, also explored by the combination of angiography and blood pressure.

Figure 4 also reveals that physiological and behavioral data streams are used together. For example, data on screen use have been employed alongside data on sleep, physical activity, communication and mobility patterns, and EMA/ESM. These combinations highlight the potential for incorporating multiple data streams within a single study, which may be collected by a single device (typically a smartphone).

To quantify the cluster structure apparent in the network visualization, we employed the link‐community detection algorithm in Ahn et al. (2010), identifying seven sets of closely related links. Figure 4b shows the co‐occurrence network grouped by color‐coded link communities. Three large communities emerge (red, blue, and green). The blue community comprises the most frequent MRI techniques‐physiological combinations, but also including physical activity, and sleep. The red community includes the most frequent MRI technique‐device combinations, focusing on devices that measure behavior. The green community integrates both uncommon fMRI‐PADs combinations, such as glucose, temperature, app use, and images, and the prevalent physical activity. Finally, four smaller communities are also detected, linking cardio‐respiratory measurements and angiography (yellow and orange), and heart rate‐related measurements (brown and black).

**FIGURE 5:**
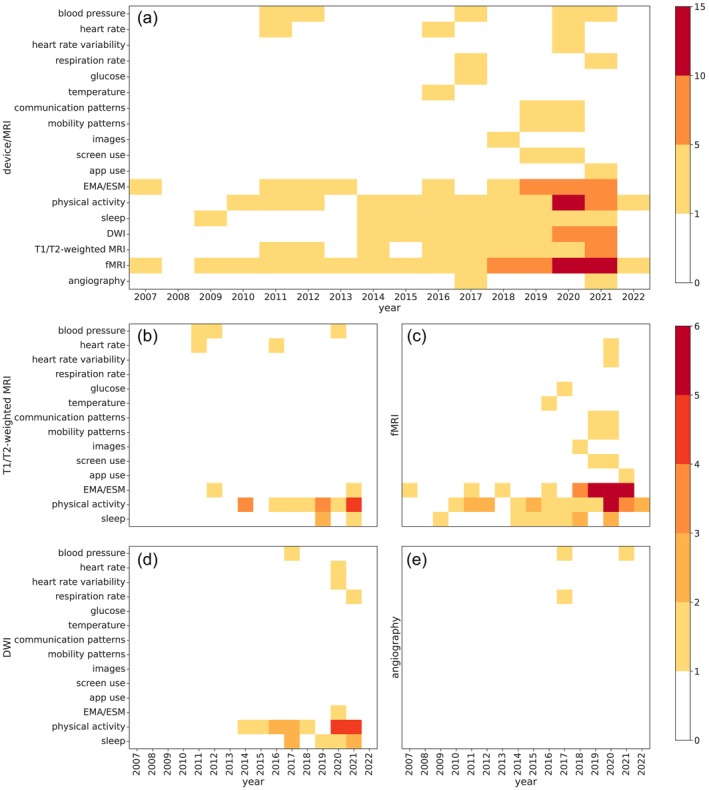
Frequency of used magnetic resonance imaging (MRI) techniques and portable automatic devices over time. (a) Colors indicate the number of papers employing a specific device and MRI technique. Panels (b)–(d)indicate the number of times a device has been used in combination with T1/T2‐weighted MRI, fMRI, DWI, and angiography.

Furthermore, we examined the frequency of MRI‐PAD use over the years to identify possible trends. Figure 5a shows that researchers started combining MRI and PAD data as early as 2007 by merging fMRI and EMA/ESM data. From 2009 onward, new variables are included such as sleep, physical activity, blood pressure, and heart rate. However, it is not until the last 5 years that we truly see a growing interest in combining MRI and PAD data.

In the fMRI case, Figure 5c shows that in 2011, a larger number of papers combining fMRI and physical activity is reported. However, it is not until 2018 that we see a clear interest in merging fMRI with physical activity, sleep, and different EMA/ESM‐measured variables. This interest peaks in 2020, when most of the papers employing fMRI are published (Figure 5a).

In 2011, T1/T2‐weighted MRI was incorporated for the first time into studies examining physiological variables derived from blood pressure and heart rate measurements. Subsequently, the focus expanded to include physical activity and sleep as the main variables of interest in human behavior (Figure 5b), peaking in 2021.

DWI appeared for the first time among the reviewed papers in 2014, in conjunction with data from physical activity. Shortly after, the interest in integrating DWI with other variables such as sleep, increased, peaking in 2020 and 2021 (Figure 5d).

Finally, only a few studies have used angiography, with its first year of publication being 2017. Interest in this modality has remained low (Figure 5e).

### 3.9 Brain areas

**FIGURE 6:**
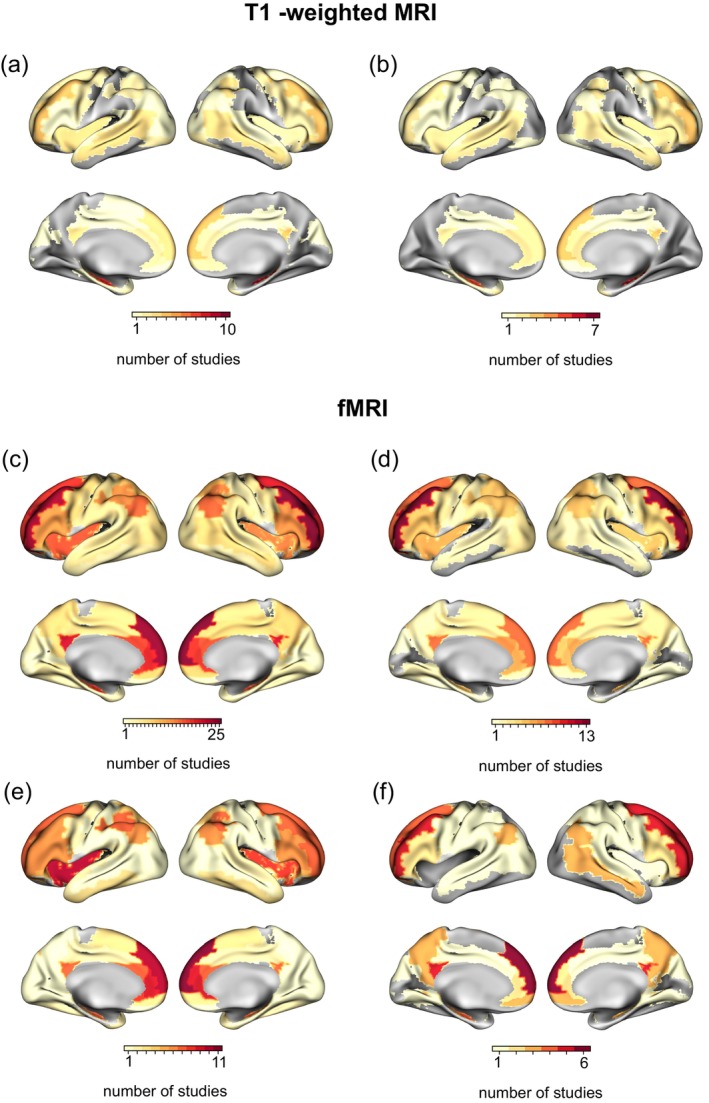
Brain areas reported across the most common magnetic resonance imaging‐portable automatic devices (MRI‐PAD) combinations. The colors represent the number of studies that reported a specific brain area as statistically significant for (a) T1‐weighted MRI alone, (b) T1‐weighted MRI and physical activity, (c) functional MRI (fMRI) alone, (d) fMRI and physical activity, (e) fMRI and EMA/ESM, and (f) fMRI and sleep.

We also visualized the list of statistically significant areas in all studies. The list includes only the areas reported as results from the MRI‐PAD combination analysis. Figure 6 shows a summary of brain areas involved across the most common MRI‐PAD combinations. Other combinations are shown in Supplementary Figures S2 and S3.

**FIGURE 7:**
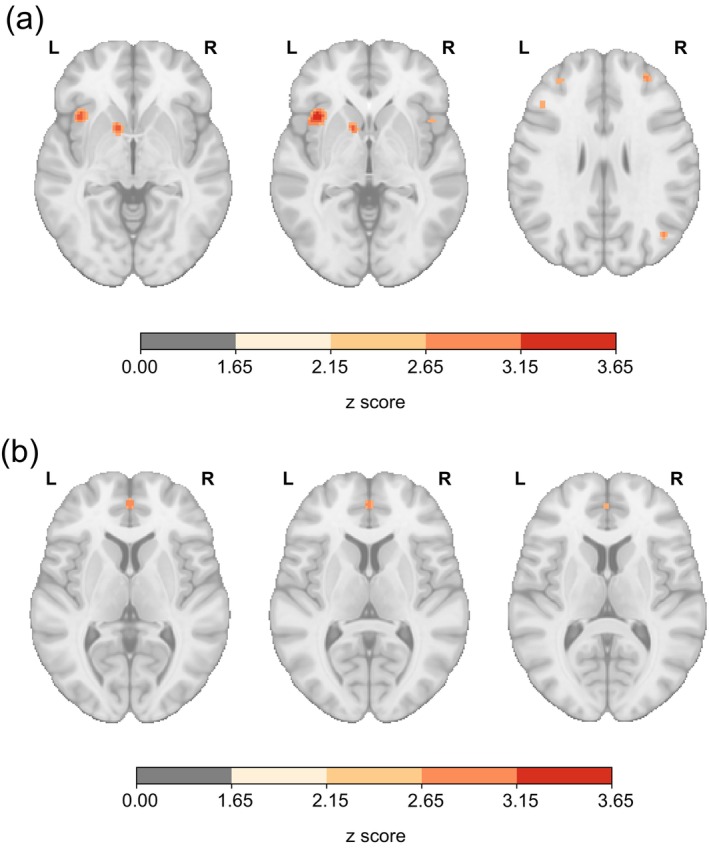
Meta‐analytical brain maps of areas reported using the most common functional magnetic resonance imaging‐portable automatic devices (fMRI‐PAD) combinations. For each study, we extracted the reported statistically significant region of interest (ROI) coordinates. Then, for each combination, we run a meta‐analysis if at least four studies reported coordinates. Significant clusters were found for studies combining (a) fMRI and EMA/ESM and (b) fMRI and sleep sensors.

From the 94 reviewed papers, we found that 44 did not provide ROI coordinates. Within this subset, seven papers omitted coordinates due to their use of whole‐brain analyses, and one paper reported no significant findings for MRI‐PAD combination analyses. The full list of coordinates per paper is in the Supplementary Table S3. Subsequently, we conducted a meta‐analysis on MRI‐PAD combinations for which there were at least four papers reporting coordinates. By selecting this number, we want to ensure that there was sufficient data to allow for meaningful statistical analysis, while also maintaining a reasonable standard of representativeness across the studies in our review. Initially, the meta‐analysis encompassed all papers corresponding to three MRI‐PAD combinations (fMRI and EMA/ESM, physical activity, and sleep). However, no significant clusters were found for studies combining fMRI and physical activity. Significant clusters for the combination of fMRI‐EMA/ESM and fMRI and sleep sensors are shown in Figure 7 and listed in the Supplementary Table S4. Following this, we performed a secondary analysis, categorizing the papers according to the type of analysis (general linear model or connectivity analysis). The unthresholded maps yielded by these meta‐analyses are shown in Supplementary Figure S4. The maps are also available in neurovault https://identifiers.org/neurovault.collection:15924.

## 4 DISCUSSION

In this study, we systematically explored and characterized research that jointly examines data from MRI and PADs used in real‐world contexts. These devices are compact, wearable, or handheld electronic devices that automatically collect data that are physiological (e.g., bodily functions) or behavioral (e.g., environmental interactions, mental states via self‐reports), with minimal user input. We automatically retrieved relevant papers from two digital libraries (PubMed and Scopus), selecting those that met predefined inclusion criteria. Subsequently, we synthesized and analyzed their methodology and results. Our analysis of the 94 selected papers shows a rapidly growing interest in developing new approaches that merge MRI techniques with PAD data. This interest is evident in the growing number of publications since 2007, the diversity of measured behaviors and their corresponding MRI combinations, and the range of sampled characteristics, such as age and illness. This trend indicates an emerging brain research paradigm shift, moving beyond traditional laboratory or clinical settings to embrace more naturalistic, real‐life contexts.

Thus far, of all PADS, smartphones have been largely covered in combination with MRI data in the work by McGowan et al. (2023), which reviewed methods to combine these data sources and to sketch a new network science framework. Our review expands the range of devices, incorporating more PAD search terms that may be of interest in the community. We also address the characteristics of included studies and discuss the brain areas they cover. Moreover, the open code provided here provides a good foundation for future researchers who would like to expand the review to other aspects. Taken together, both reviews point out to the growing interest in combining MRI and PADs for the study of brain–behavior relationships.

### 4.1 The most common combinations are fMRI, T1/T2‐weighted MRI, and DWI with physical activity, EMA, and sleep

The graph analysis revealed a cluster of the most common MRI‐PAD combinations (Figure 4a). This cluster featured fMRI, T1/T2‐weighted MRI, and DWI on the MRI side, which is not surprising considering their extensive use in brain research. Within this cluster, the MRI techniques were frequently paired with PADs that collect information on physical activity, sleep patterns, and thoughts and behaviors through EMA/ESM.

The inclusion of physical activity and sleep patterns in this cluster can be attributed to two key factors. First, their measurement is relatively simple and cost‐effective due to the widespread use of accelerometers, which are the most frequently used device in the selected studies (Table 4). This is partly because they have been widely integrated into devices such as actigraphs and smartphones. Finally, the well‐established clinical relevance of these markers makes them particularly attractive for researchers and clinicians, as the findings can be readily translated into actionable steps to improve health outcomes.

Despite their shared relevance, sleep studies are less represented than physical activity, which may be partially explained by PAD limitations in sleep staging measurement (Imtiaz, 2021). Because of these, sleep measurements are mostly restricted to sleep duration, onset, and offset. Therefore, some researchers prefer the accuracy of polysomnography to the portability of PADs. Nevertheless, recent advances in portable devices, such as ear‐electroencephalography and tattoo‐like electrodes, are likely to improve the reliability of signals collected during sleep outside a sleep laboratory (Casson, 2019).

Finally, EMA/ESM also draws noticeable interest, particularly in social‐context research. The use of EMA/ESM precedes the advent of smartphones, but it has adapted well to various technologies over time, including integration within smartphones themselves. This makes EMA/ESM the oldest methodology to be utilized by PADs. Although EMA/ESM requires user input and thus imposes a higher burden compared to other passive methods, its versatility and adaptability continue to draw significant attention from researchers. This is evident in the robust connection between fMRI and EMA in Figure 4a.

### 4.2 Combination of MRI and physiological measurements is feasible, but underexplored

The graph analysis revealed other intriguing patterns, with three distinct groups identified through link community detection (Figure 4b). These encompass the previously mentioned cluster (red), a group of less well‐connected nodes (green), and a cluster focused on common physiological measurements (blue). This last cluster also includes common behavioral measurements (physical activity and sleep) that are known to greatly affect human physiology (Atkinson & Davenne, 2007; Janssen et al., 2020).

Notably, the blue group demonstrates the feasibility of collecting physiological measurements in naturalistic settings using PADs and indicates a promising direction for future research. In particular, the advances in photoplethysmography and its implementations in smartwatches have facilitated the reliable collection of physiological data in real‐time (Fuller et al., 2020). As cardiac function is significantly influenced by the brain's central autonomic system (Silvani et al., 2016), expanding research on brain–heart interactions is vital. Furthermore, in the clinical context, analyzing brain–heart interactions in naturalistic settings offers promising tools for the prognosis of neurological disorders (Boots et al., 2019; Silvani et al., 2016).

### 4.3 There is an increasing interest in combining MRI and PAD technologies to study brain–behavior relationships

Research merging MRI and PAD data has grown in volume and diversity since the first publication in 2007 (Figure 5). In the early years, a few studies focused on combining fMRI with physical activity, EMA/ESM, and sleep. Since then, the number of published papers has increased and more MRI techniques have been incorporated. For example, there has been a rise in the use of DWI in the last 3 years and the inclusion of angiography in the past 5 years.

The growing diversity in research is also demonstrated by the integration of advanced PADs that can simultaneously measure complex behaviors from multiple data streams, such as mobility and communication patterns. Interestingly, these PADs were included in studies 4 years after the first papers on digital phenotyping was published (Jain et al., 2015; Torous et al., 2016).

Combining MRI and PAD data presents unique challenges and potential measurement issues. One key challenge is integrating these two data types, given their inherent differences in format, resolution, and timing. The integration of smartphone‐MRI data, as discussed in McGowan et al. (2023), addresses some of these challenges proposing a new framework that is adaptable to other PADs beyond smartphones (e.g., fitness trackers). Similar works will be needed in the future, as research shifts from observational to experimental approaches.

Signal artifacts and noise in PAD data pose another substantial challenge, as factors such as motion artifacts, variable environmental conditions, battery life, user engagement, or electronic interference can affect data quality (Böttcher et al., 2022; Onnela, 2021). PAD accuracy and reliability is still a concern, especially since issues like data missingness or quality are not comprehensively reported in current MRI‐PAD studies. In our review, 54 studies did not comment on PAD data missingness or quality, underscoring the urgent need for more transparent reporting practices to guide and improve the design of future research.

In MRI, movement artifacts, particularly head motion, significantly impact data accuracy (Ciric et al., 2018; Gilmore et al., 2021; Hedges et al., 2022; Oldham et al., 2020; Power et al., 2012). Our review of the literature revealed that in the period from 2007 to 2020, 5 fMRI studies, 10 T1‐weighted MRI studies, and 8 DWI studies did not report any measures for correcting head motion, suggesting that this remains an ongoing concern. Notably, in fMRI research, where head motion has been closely scrutinized (Ciric et al., 2018; Lynch et al., 2021; Power et al., 2012), 20 papers specifically excluded data based on head movement parameters, demonstrating a heightened awareness of this factor in certain areas of MRI research.

### 4.4 The medial prefrontal cortex, hippocampus, and dorsolateral frontal regions are most commonly associated in studies merging MRI and PAD data

The visualization of significant brain regions reported in the selected studies (Figure 6) highlights the medial prefrontal cortex, hippocampus, and dorsolateral frontal regions as the most frequently reported areas. Given their pivotal role in working memory and executive functions such as goal‐directed behavior (Friedman & Robbins, 2022), it is expected to find a strong correlation between these regions and behavior measurements derived from real‐world PADs. Interestingly this pattern does not seem to heavily depend on whether the association between brain activity and PADs is due to EMA/ESM, physical activity, or sleep sensors.

The hippocampus and prefrontal cortex play critical roles in various behavioral and cognitive functions, such as memory consolidation, decision‐making, and emotional regulation. Dysfunction within this network is associated with several psychiatric disorders, such as schizophrenia, major depressive disorder, and post‐traumatic stress disorder (Euston et al., 2012; Sigurdsson & Duvarci, 2016).

Complementary to the brain visualizations, the meta‐analyses show significant clusters in the insula, pallidum, and anterior cingulate cortex. Given most of the studies combining fMRI and EMA/ESM focused on measuring mood, affect, and self‐control, it is expected to find areas related to self‐awareness, emotion processing, reward, and decision‐making (Gu et al., 2013; K. S. Smith et al., 2009). While behavioral measurements from PADs might only reflect a correlational relationship with the functionality of these brain areas, they can serve as a valid proxy for cost‐effective diagnostics that do not need to rely on MRI. Additionally, they can be used as reliable outcome measures in future intervention studies and behavioral therapy.

### 4.5 Age is not a constraint, but adherence may be

Although continuous monitoring in elderly populations might be challenging due to their declining physiological and psychological conditions (Kekade et al., 2018), this review shows that age is not a constraint for including elderly individuals in studies that simultaneously collect brain MRI and PAD data (Figure 2). Supporting this finding, (Ahmad et al., 2020; Ma et al., 2021) report that older adults are willing to use technology when they perceive it as useful and easy to use. However, willingness to adopt unfamiliar technologies can be influenced by age (Ma et al., 2021). Similarly, research indicates that children are also receptive to using portable devices for health research purposes (Dimitri, 2019; Nadal et al., 2020).

While chronological age is not an obstacle in the reviewed studies, other factors may affect a subject's participation. One factor is the willingness of subjects to participate in PAD data collection protocols. We found two studies that reported subjects who refused to enroll in data collection with physical activity PADs (Ahmed et al., 2017; Kokotilo et al., 2010). Although this is a small proportion, being aware of these cases can help researchers factor in potential difficulties during the recruiting phase. This underscores the necessity of including patient and public involvement activities in clinical study designs deploying PADs (Hassan et al., 2017).

Another crucial factor is participant exhaustion, which can be influenced by the chosen PAD and sampling strategy. In general, reviewed studies employed PADs for periods ranging from 1 to 243 days, with most studies using devices for approximately 10 days (Figure 3d). Blood pressure PADs were employed for the shortest time due to their cumbersome characteristics, limitations (Dadlani et al., 2019; Ling et al., 2020), and current clinical practices (Pena‐Hernandez et al., 2020). In contrast, fitness trackers are well suited for longer research studies, as they collect data passively and are common in everyday use (Evenson et al., 2015). Finally, EMA/ESM and other active data collection strategies may be associated with some burden for subjects, posing a challenge in maintaining engagement (Onnela, 2021). Financial incentives can boost engagement over short periods, but this may not be feasible for large cohorts or noncommercial research. Ultimately, striking a balance between accurate data collection and participant comfort is key for the success of studies involving PADs.

While extending data collection time may benefit many studies, it is also crucial to consider how it may affect data quality. In our sample, the number of subjects excluded due to poor PAD data quality is comparable to those discarded for MRI‐related issues or other reasons (e.g., medical conditions or phobias). However, in the reviewed studies, reported reasons for discarding PAD data include insufficient, inconsistent, or corrupted data, user refusal to use the device, technical problems, or failure to capture the desired effect. As PADs are frequently deployed blindly in natural settings, data inadequacies only become apparent after data collection has ended. This highlights the need for improvements in data collection systems and PADs themselves.

### 4.6 Dynamic relationships between brain and behavior

While cross‐sectional designs have significantly advanced our knowledge of brain–behavior relationships, translating their results remains challenging. Here, we identified several research papers that have successfully demonstrated the feasibility and benefits of integrating PAD data with MRI using various study designs, such as longitudinal, controlled trials, and interventions (Table 3). This promising approach could lead to a better understanding of how real‐life factors like sleep influence brain responses over different time scales (e.g., hours, days, months). Additionally, the inclusion of PAD data may help elucidate what is currently considered “noise” or error signals in MRI analysis, revealing ecologically relevant variables and providing insights into the intra‐ and inter‐individual differences.

### 4.7 Clinical inclusion

Notably, almost half of the reviewed research papers include a group diagnosed with an illness (Table 3). Naturally, as the brain is the primary focus, most attention is directed toward psychiatric and neurological disorders. However, the inclusion of other illnesses indicates a shift in how researchers and clinicians approach disease investigation. These studies demonstrate the importance of brain–behavior dynamics in various physiological aspects beyond the brain itself.

This broader perspective extends beyond focusing solely on the organ where symptoms appear, to include interactions between different body systems. Many studies could benefit from the combined analysis of MRI and PAD data. For example, cerebrovascular perfusion MRI data may require normalization to baseline physiological measurements in stroke research (Boots et al., 2019). In fact, the BOLD response might be significantly shaped by physiological changes. Therefore, to enhance the accuracy of fMRI studies, long‐term physiological data may be necessary for post‐processing results and interpretations (Shmueli et al., 2007).

In the behavioral domain, other examples may include psychiatric imaging studies, where monitoring changes in behavioral patterns (Huckvale et al., 2019) or controlling for symptom severity during a study (Lepage et al., 2020) are vital. In these cases, the combined use of PAD and MRI data could enable estimation of causal links between MRI measurements and symptoms, as well as the detection of early warning signs (Wichers et al., 2020).

Finally, since MRI tests are expensive and not prescribed for every individual, understanding the connections between real‐world behavior and physiology measured by PADs and MRI brain data becomes crucial. By monitoring a patient's behavior using PADs, we could determine the need for more specialized MRI tests based on the collected PAD data, thus optimizing resources and clinical management.

### 4.8 Limitations and future work

There are several limitations to consider. First, the review only covered published studies, excluding protocols, books, conference abstracts, dissertations, and theses. Future reviews could also encompass these. Second, we searched for articles in only two digital databases that allowed for automatic retrieval of papers. Future research may extend the search to other databases. Third, only one person fully read and classified the papers. Future efforts could involve more reviewers examining the papers and employing quality control to verify the extracted information.

The sample of papers reviewed here has some notable limitations that warrant further considerations. First and foremost, the majority of the reviewed research is observational, and therefore correlative and exploratory in nature. As a result, one should be cautious about inferring causative mechanisms based on these observations. Only few papers (Bakker et al., 2019; Balbim et al., 2021; Burzynska et al., 2017; Kalafatakis et al., 2021; Michielse et al., 2020; Morris et al., 2022; Rodriguez‐Ayllon et al., 2020; Servaas et al., 2019; J. L. Smith et al., 2021) have employed controlled trials or interventional studies that test specific hypotheses. Given the growing interest in integrating MRI and PAD data, future research should increasingly emphasize experimental approaches. This shift is crucial for gaining a deeper and more detailed understanding of the interplay between brain function and behavior.

Additionally, while we visually summarized statistically significant brain areas as reported in the studies, these maps should be interpreted with caution. The visual summaries serve an informative purpose, highlighting trends in results from specific MRI and PAD combinations, helping researchers by identifying the relevant literature in the context of brain areas of interest. Furthermore, the visual summaries offer a valuable alternative in situations where a great portion of the studies lack reported coordinates, hindering meta‐analysis due to insufficient data. As an example, the EMA/ESM method was not categorized into functional domains due to its broad adaptability—researchers often customize questionnaires to suit their specific study needs (refer to, e.g., Horstman et al., 2022 and Sequeira et al., 2021); categorization into functional categories would require an analysis beyond this review's scope. To provide further context, we include the EMA‐measured constructs in Table 2.

Care is also advised when interpreting maps, such as those correlating physical activity and MRI modalities. For example, while sensorimotor areas might be expected to feature prominently in studies using PADs with accelerometers, the specifics of the studies must be considered. Some studies investigated connectivity patterns in selected ROIs excluding sensorimotor areas, employed whole‐brain analysis, or used MRI paradigms to target specific cognitive functions. However, specific studies focusing on sensor regions, such as Kokotilo et al. (2010) that analyzed left arm movement in stroke patients, did find significant activity in these areas.

## 5 CONCLUSIONS

Combining neuroimaging with personalized behavioral and physiological information is becoming crucial for the development of precision medicine (Scala et al., 2023). Traditionally, neuroimaging studies have tended to only focus on late‐stage clinical manifestations of disease, with little consideration for the coincident underlying physiology and behavior that likely influences image interpretation (Hampel et al., 2023). Therefore, combining MRI and PAD data may sharpen and refine future methods and models for interpreting imaging data. Alternately, the interpretation of a subject's behavioral or physiological activity may be shaped by detailed knowledge of concurrent brain activity, for example, during optimal athletic performance (Furrer et al., 2023). Finally, predictive medicine models within the growing health “omics” revolution are becoming ever more reliant on large data sets from concurrent multimodal sources. Consequently, integrating MRI data with many types of PAD data will be necessary to help harness the full potential of multimodal artificial intelligence in future health technologies (Acosta et al., 2022).

## AUTHOR CONTRIBUTIONS

NMEAH and EG conceptualized the idea. EG, NMEAH, and JS supervised the methodology. EG and JS supervised the analysis. AMT, EG, and NMEAH developed the screening methodology and jointly screened the abstracts, reaching consensus in cases of uncertainty regarding paper inclusion. AMT wrote the software for the automatic retrieval of abstracts and read the papers, extracting relevant data, analyzing the information, and creating visualizations. EG generated the brain maps visualizations and conducted quality control on them. All authors contributed to the writing of the final manuscript.

## CONFLICT OF INTEREST STATEMENT

The authors have declared no competing interests.

## Supporting information


**FIGURE S1:** Rating distribution of graders. Each author assigned a score of 1 for inclusion, −1 for exclusion, or 0 for uncertainty. A paper was rejected if at least two graders graded it’‐1′. (A) The cumulative sum between the grader's independent score was computed, showing that more than half of the papers were rejected unanimously by the three reviewers, given by the skewed distribution. This skewness is also found in the graders' independent distributions: (B) EG, (C) AMT, and (D) NMEAH.

**FIGURE S2:** Brain areas reported across the most common T1/T2‐weighted MRI‐PAD combinations. The colors represent the number of studies which reported a specific brain area as statistically significant for: (A) sleep, (B) EMA/ESM, (C) heart rate, and (D) blood pressure.

**FIGURE S3:** Brain areas reported across the most common fMRI‐PAD combinations. The colors represent the number of studies which reported a specific brain area as statistically significant for: (A) application use, (B) screen use, (C) images, (D) communication patterns, (E) mobility patterns, (F) temperature, (G) glucose, (H) heart rate, and (I) heart rate variability.

**FIGURE S4:** Meta‐analysis based on the most common fMRI‐PAD combinations. For each study, we extracted the statistically significant ROI coordinates for analysis using MRI‐PAD combinations. For each combination, we run a meta‐analysis if at least four studies reported coordinates. We run analysis for fMRI and (A) all PADs, (B) EMA/ESM, (C) physical activity, and (D) sleep regardless of their analysis method (GLM or connectivity). In addition, we run analysis for the studies reporting fMRI and (E) all PADs, (F) EMA, (G) physical activity, and (H) sleep using only GLM methods. We also run analysis for the studies reporting fMRI and (I) all PADs, (J) EMA, (K) physical activity, and (L) sleep using only connectivity methods. Finally, we run an analysis for the (M) T1‐weighted and all PADs combinations for both connectivity and GLM methods. Other combinations of T1‐weighted‐PADs did not surpass the four papers threshold we established. All maps are unthresholded.

**DATA S1.** Tables.

**DATA S2.** Supporting Information.

## Data Availability

Data sharing is not applicable to this article as no new data were created or analyzed in this study.
