# Supplementary figures and images for: Neuroscience meets behavior: A systematic literature review on magnetic resonance imaging of the brain combined with real‐world digital phenotyping
Source: Hum Brain Mapp. 2024 Mar 4;45(4):e26620. doi: 10.1002/hbm.26620 (PMC10911114; doi:10.1002/hbm.26620)

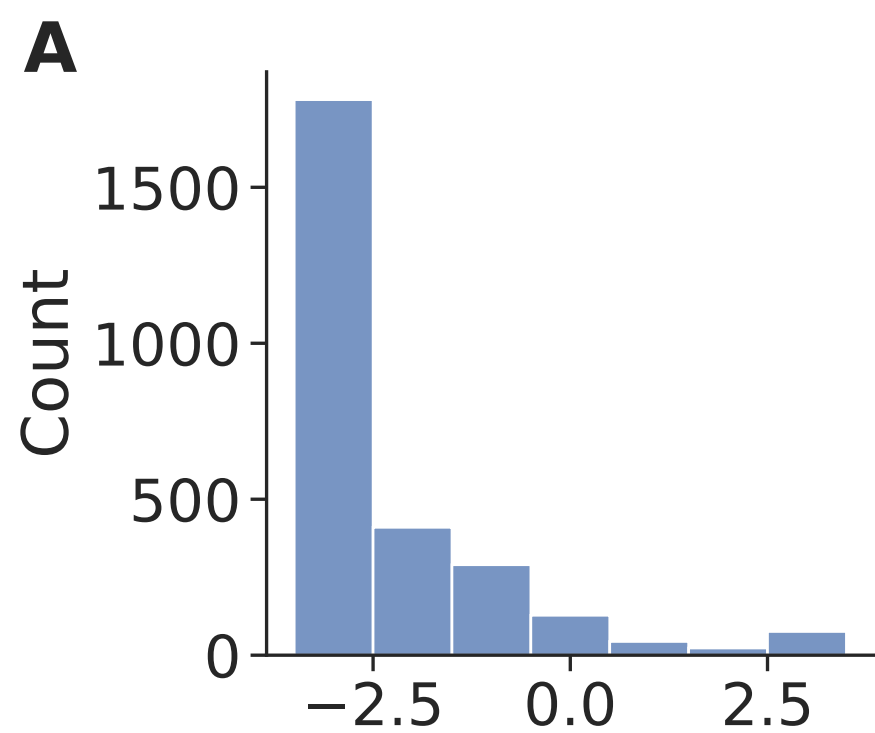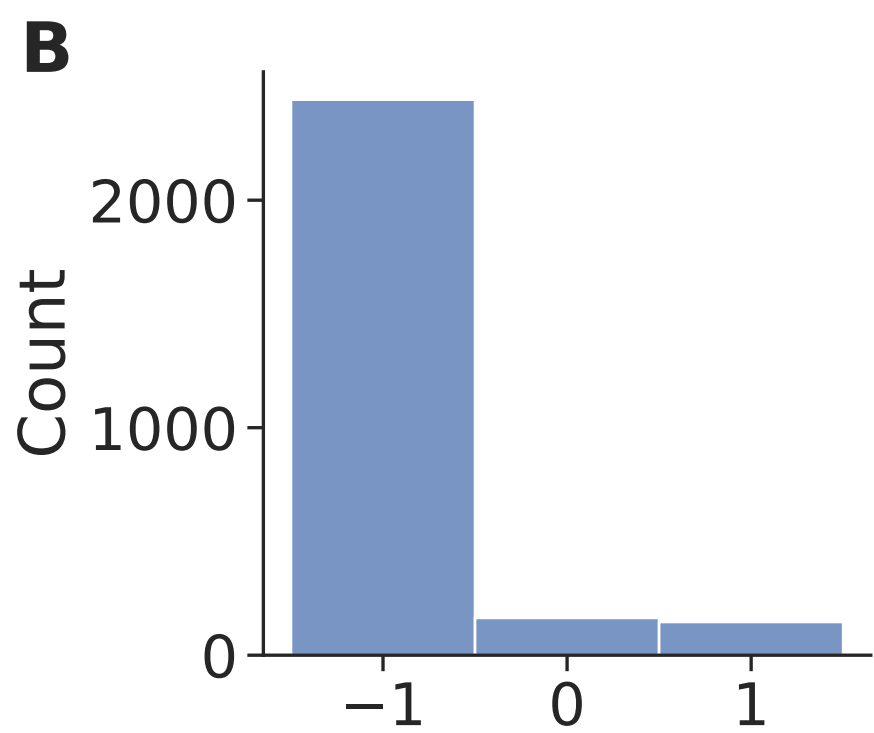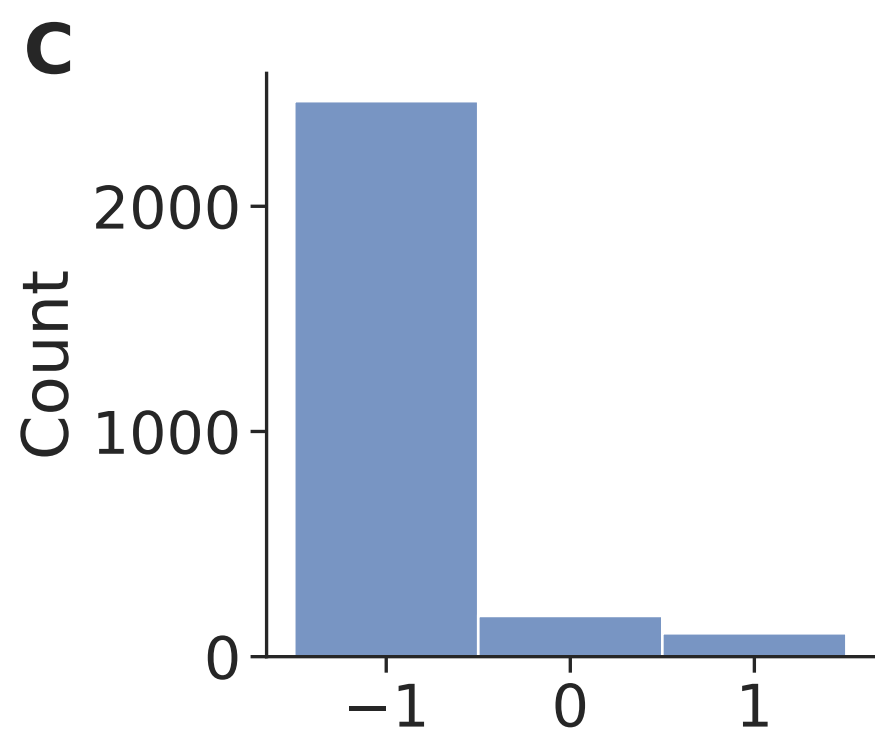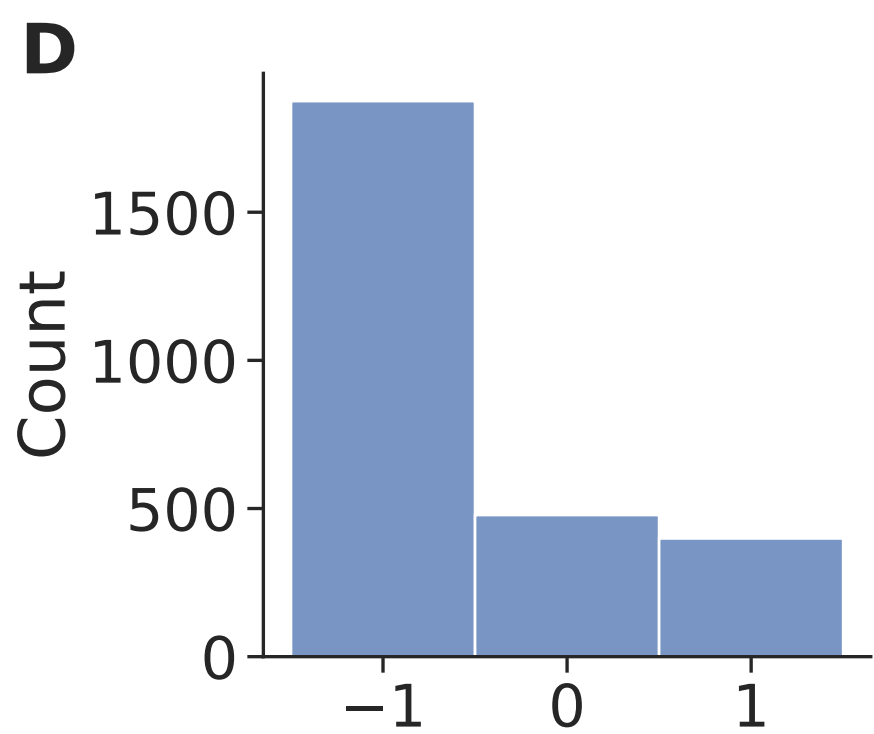

Supplement: Supplementary file 1 — FIGURE S1: Rating distribution of graders. Each author assigned a score of 1 for inclusion, −1 for exclusion, or 0 for uncertainty. A paper was rejected if at least two graders graded it’‐1′. (A) The cumulative sum between the grader's independent score was computed, showing that more than half of the papers were rejected unanimously by the three reviewers, given by the skewed distribution. This skewness is also found in the graders' independent distributions: (B) EG, (C) AMT, and (D) NMEAH. [file HBM-45-e26620-s002.pdf]

A

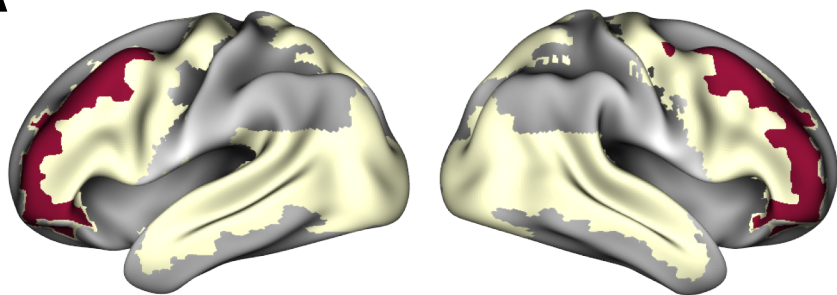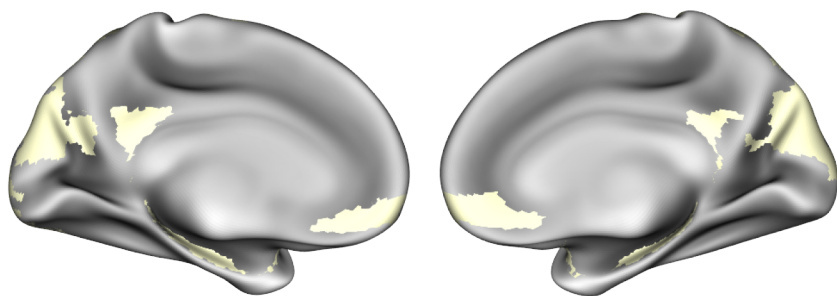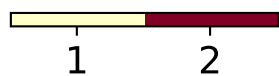

number of studies

B

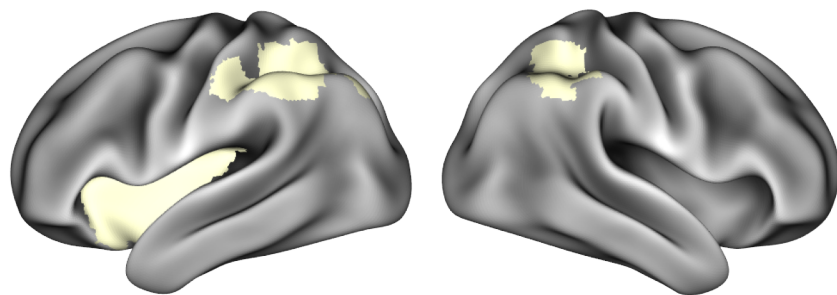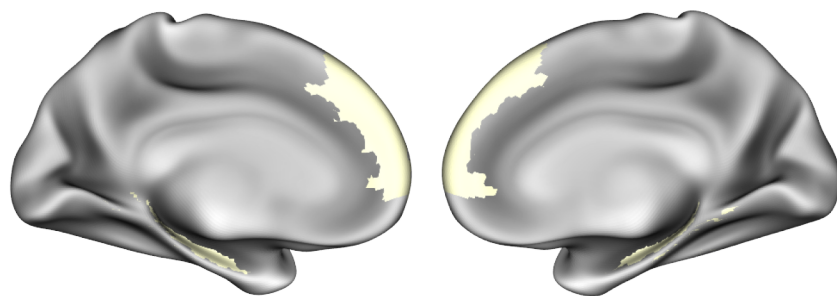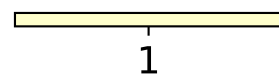

number of studies

C

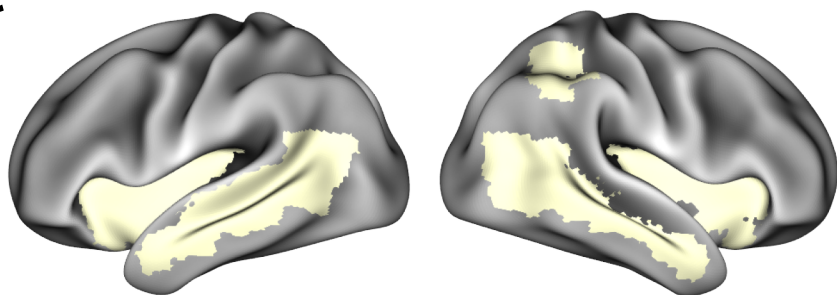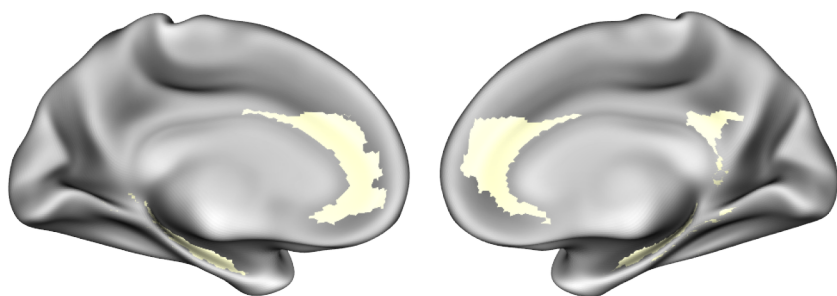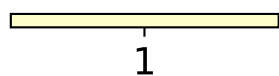

number of studies

D

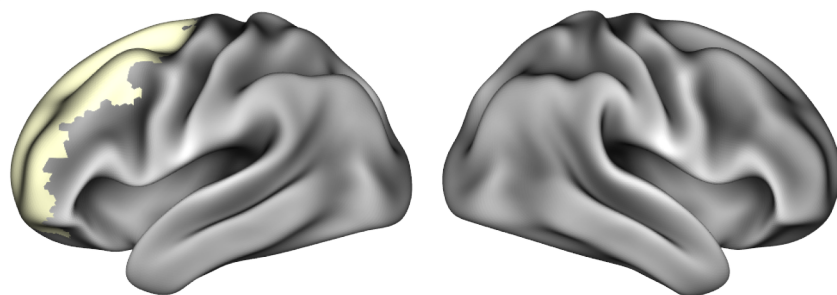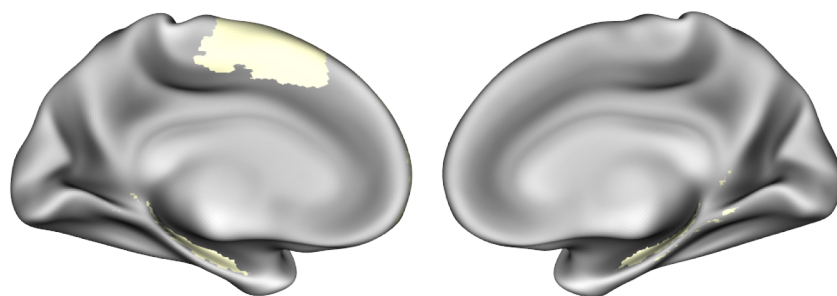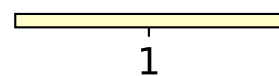

number of studies

Supplement: Supplementary file 2 — FIGURE S2: Brain areas reported across the most common T1/T2‐weighted MRI‐PAD combinations. The colors represent the number of studies which reported a specific brain area as statistically significant for: (A) sleep, (B) EMA/ESM, (C) heart rate, and (D) blood pressure. [file HBM-45-e26620-s001.pdf]

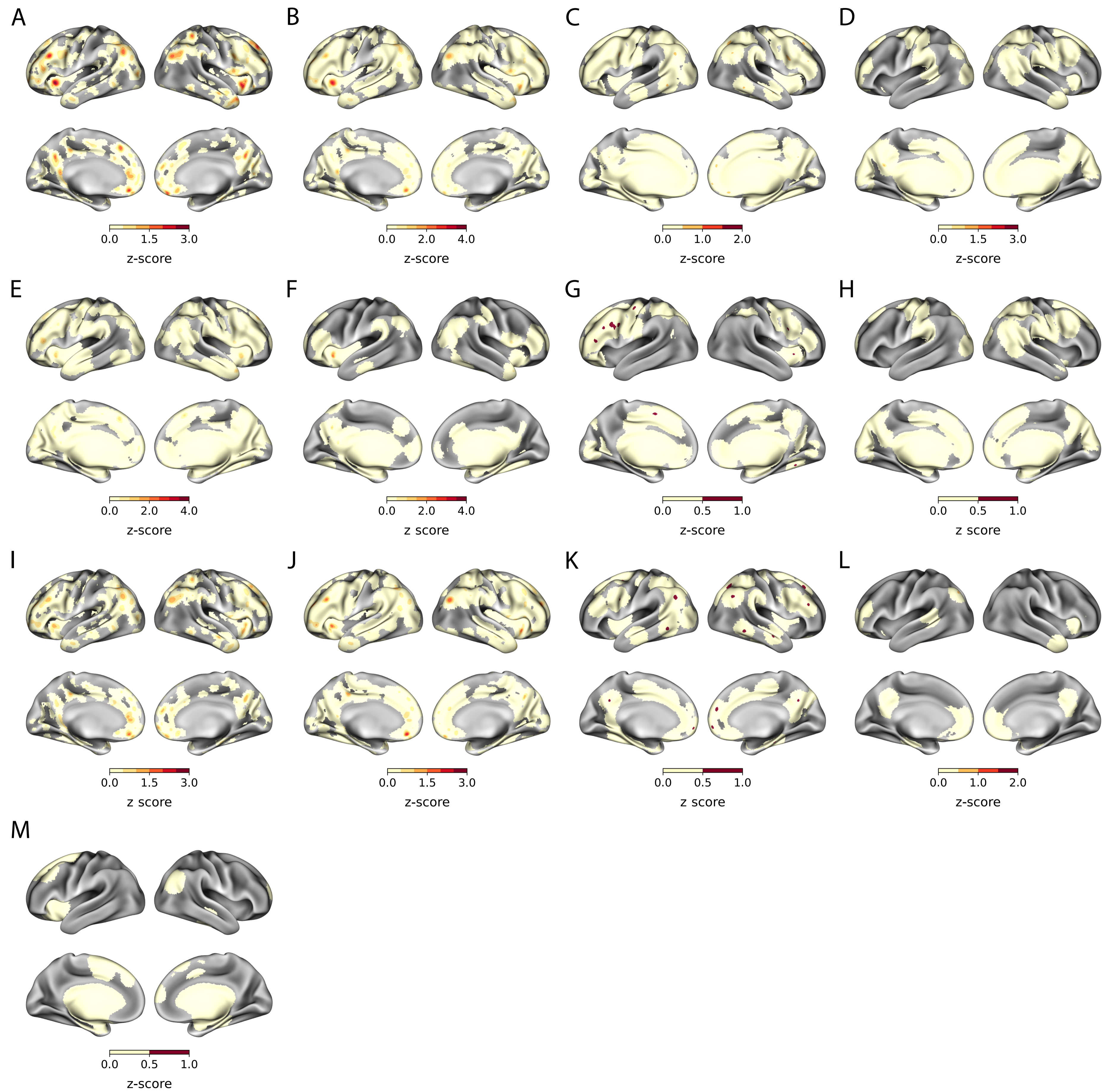

Supplement: Supplementary file 4 — FIGURE S4: Meta‐analysis based on the most common fMRI‐PAD combinations. For each study, we extracted the statistically significant ROI coordinates for analysis using MRI‐PAD combinations. For each combination, we run a meta‐analysis if at least four studies reported coordinates. We run analysis for fMRI and (A) all PADs, (B) EMA/ESM, (C) physical activity, and (D) sleep regardless of their analysis method (GLM or connectivity). In addition, we run analysis for the studies reporting fMRI and (E) all PADs, (F) EMA, (G) physical activity, and (H) sleep using only GLM methods. We also run analysis for the studies reporting fMRI and (I) all PADs, (J) EMA, (K) physical activity, and (L) sleep using only connectivity methods. Finally, we run an analysis for the (M) T1‐weighted and all PADs combinations for both connectivity and GLM methods. Other combinations of T1‐weighted‐PADs did not surpass the four papers threshold we established. All maps are unthresholded. [file HBM-45-e26620-s006.pdf]
