# Supplementary material for: Neuroscience meets behavior: A systematic literature review on magnetic resonance imaging of the brain combined with real‐world digital phenotyping
Source: Hum Brain Mapp. 2024 Mar 4;45(4):e26620. doi: 10.1002/hbm.26620 (PMC10911114; doi:10.1002/hbm.26620)
Supplement: Supplementary file 3 — FIGURE S3: Brain areas reported across the most common fMRI‐PAD combinations. The colors represent the number of studies which reported a specific brain area as statistically significant for: (A) application use, (B) screen use, (C) images, (D) communication patterns, (E) mobility patterns, (F) temperature, (G) glucose, (H) heart rate, and (I) heart rate variability. [file HBM-45-e26620-s004.pdf]

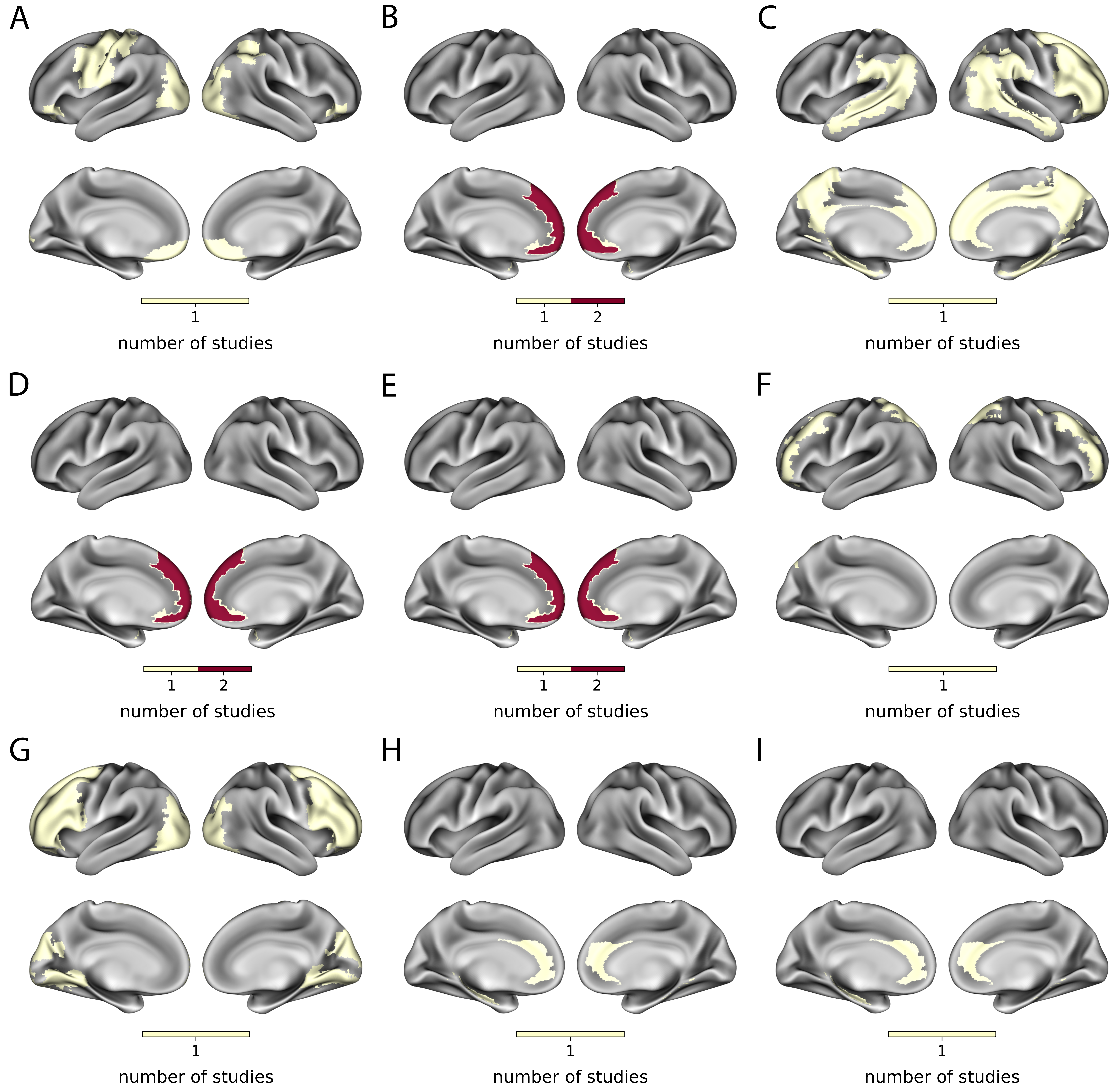
A
1
number of studies
B
1
2
number of studies
C
1
number of studies
D
1
2
number of studies
E
1
2
number of studies
F
1
number of studies
G
1
number of studies
H
1
number of studies
I
1
number of studies
